# Dissecting the Single-Cell Transcriptome Network of Immune Environment Underlying Cervical Premalignant Lesion, Cervical Cancer and Metastatic Lymph Nodes

**DOI:** 10.3389/fimmu.2022.897366

**Published:** 2022-06-24

**Authors:** Chunbo Li, Keqin Hua

**Affiliations:** ^1^ Department of Obstetrics and Gynecology, Obstetrics and Gynecology Hospital of Fudan University, Shanghai, China; ^2^ Shanghai Key Laboratory of Female Reproductive Endocrine Related Diseases, Shanghai, China

**Keywords:** cervical cancer, single-cell sequencing, tumor microenvironment, squamous cell carcinoma, immune cell

## Abstract

Cervical cancer (CC) is one of the most common malignancy in women worldwide. It is characterized by a natural continuous phenomenon, that is, it is in the initial stage of HPV infection, progresses to intraepithelial neoplasia, and then develops into invasion and metastasis. Determining the complexity of tumor microenvironment (TME) can deepen our understanding of lesion progression and provide novel therapeutic strategies for CC. We performed the single-cell RNA sequencing on the normal cervix, intraepithelial neoplasia, primary tumor and metastatic lymph node tissues to describe the composition, lineage, and functional status of immune cells and mesenchymal cells at different stages of CC progression. A total of 59913 single cells were obtained and divided into 9 cellular clusters, including immune cells (T/NK cells, macrophages, B cells, plasma cells, mast cells and neutrophils) and mesenchymal cells (endothelial cells, smooth muscle cells and fibroblasts). Our results showed that there were distinct cell subpopulations in different stages of CC. High-stage intraepithelial neoplasia (HSIL) tissue exhibited a low, recently activated TME, and it was characterized by high infiltration of tissue-resident CD8 T cell, effector NK cells, Treg, DC1, pDC, and M1-like macrophages. Tumor tissue displayed high enrichment of exhausted CD8 T cells, resident NK cells and M2-like macrophages, suggesting immunosuppressive TME. Metastatic lymph node consisted of naive T cell, central memory T cell, circling NK cells, cytotoxic CD8+ T cells and effector memory CD8 T cells, suggesting an early activated phase of immune response. This study is the first to delineate the transcriptome profile of immune cells during CC progression using single-cell RNA sequencing. Our results indicated that HSIL exhibited a low, recently activated TME, tumor displayed immunosuppressive statue, and metastatic lymph node showed early activated phase of immune response. Our study enhanced the understanding of dynamic change of TME during CC progression and has implications for the development of novel treatments to inhibit the initiation and progression of CC.

## Introduction

Cervical Cancer (CC) is one of the most common malignancies in women worldwide, accounting for 7.5% of all female cancer deaths (1). A large majority (about 85%) of the global burden occurs in the less developed regions, where it accounts for almost 12% of all female cancers (2). The most common subtype of CC, especially in HPV infection, is cervical squamous cell carcinoma (SCC) (3). CC is a chronic complex disease, which is caused by genetic factors and external environmental effects. Human papillomavirus (HPV) has been identified as the main factor leading to CC (4). With the continuous infection of HPV, various oncogenic genome, transcriptome, and epigenetic changes accumulate in epithelial cells in a stepwise manner, affecting various signal pathways driving CC (5). Therefore, the range of continuous pre-invasive stages before CC makes it a feasible model to study the early evolution of cancer.

Importantly, CC consists of is a complex tumor microenvironment (TME), including cells (tumor-infiltrating immune cells and stromal cells), chemical (chemokines and cytokines), and physical components (extracellular matrix) (6). The regulation of immune response, remodeling of extracellular matrix and tumor angiogenesis essentially determine the invasiveness of cancer (7). Therefore, the understanding of comprehensive tumor microenvironment characteristics, can provide more accurate patient stratification. Traditional bulk omics analysis has inherent limitations in providing accurate information of individual cells in highly mixed TME (8). Single-cell sequencing of patient tissues is becoming an essential tool to evaluate the clinical relevance of individual cell types in tumors (9). In the specific context of the CC, our prior study has established the transcriptional profiles of normal and tumor cells types in CC using single-cell approaches (10). However, there is no study to explore the change characteristic of TME during CC progression.

In this study, we first analyzed the changes of transcription status of immune cells and stromal cells during CC progression by single-cell RNA transcription, including normal, HSIL, tumor and lymph node metastatic tumor tissues. This study will provide a deeper understanding of the cellular characteristics of TME during CC progression, and help to develop novel therapies to inhibit the initiation and progression of CC.

## Method

### Enrollment of Patients

The study was approved by the ethics committee of our hospital. We have complied with all relevant codes of ethics. Written informed consent was obtained from all patients in this study. All patients did not receive anti-tumor treatment before surgery. Samples were collected from patients diagnosed with HSIL or tumor. Finally, the tumor stages and grades were finally determined by standard histopathology.

### Tissue Processing for Single-Cell Suspension

Tissue samples (2-3 mm3) from patients were rinsed with phosphate-buffered saline (PBS) on ice. Subsequently, each sample was placed in the 500 µL dissociation medium containing 0.5 mg/mL collagenase IV (Sigma) and 1 mg/mL DNAse I (Sigma) in RPMI-1640 (ThermoFisher Scientifific). The samples were chopped in dissociation medium and then incubated at 37°C for 30 min, rotating manually every 10 minutes. Then, 1 mL cold RPMI-1640 containing 10% fetal bovine serum (FBS, ThermoFisher Scientifific) was added and each sample was filtered using 70-µm nylon mesh (ThermoFisher Scientifific). Followed by another filtration using 40-µm nylon mesh (ThermoFisher Scientifific). Samples were centrifuged with 300×g for 5 min, and the supernatant was discarded. Single cells were resuspended in 1 mL ACK lysis buffer (ThermoFisher Scientifific) and incubated for 5 min. 5 mL cold RPMI-1640 containing 10% FBS was added and the cell mixture was centrifuged at 300×g for 5 min at 4°C. Single-cell particles were resuspended in PBS without calcium and magnesium ions to achieve a density ≤1000 cells/µL.

### Single-Cell Sequencing

Cell suspensions were loaded on a Chromium Single Cell instrument (10x Genomics) to produce single-cell Gel Beads-in-emulsion (GEMs). Then, the single-cell RNA sequence library was estimated using version 1 Chromium Single-Cell 30 Library, Gel Bead & Mutiplex Kit (10x Genomics). Sequencing was performed on the Illumina NextSeq500, containing a length of 59 bp. Cell Ranger (version 3.0.1) was used with default parameters to perform sample multiplexing, barcode processing, and single-cell gene unique molecular index counting (https://software.10xgenomics.com/single-cell/overview/welcome) (11).

### QC and Cell Type Identification

Seurat (version 3.0.1) was used for the procession QC (12). Cells with < 200 unique molecular identifiers (UMIs) or mitochondrion-derived UMI counts > 10% in a single cell were considered low-quality cells and removed. The top 30 principal components, and the first 2,000 variable genes, were used in this process. Then, the inflow of UMI count and the percentage of mitochondrion-derived UMI counts were regressed by the ScaleData function. Subsequently, Seurat’s findclusters function was used to identify the main cell clusters. The Louvain clustering algorithm embedded in Seurat software was used for clustering, and the t-distributed stochastic neighbor embedding (t-SNE) method was used to visualize the clustering results. For any cell cluster, it was mainly identified because of the differences of cell cycle and did not participate in downstream analysis. To accurately annotate cell types, we manually collated genetic markers for each cell type. In particular, most of the markers used to distinguish different cell types were retrieved from the Cell Markers database (https://www.labome.com/method/Cell-Markers.html) (13). Other marker genes came from published papers.

### DEGs and GSVA

The specific markers for each cluster were identified by performing the FindAllMarkers function on the standardized expression data in the Seurat software package (only.pos =T, min.pct = 0.25) (14). Genes with adjusted P-value < 0.05 were considered statistical significance for KEGG and GO enrichment analysis. The ClusterProfiler package (version 3.14.3) was used to enrich and analyze cluster-specific biomarker genes (15). GSEA was performed with MSigDB gene sets to determine the differential pathways (16). The full gene lists of T cells signature (including the cytotoxic, exhausted, regulatory, naive, and costimulatory activity of T cells) were extracted from the published report by Chung et al. (17).

### Trajectory Analysis

Trajectory analysis of CD8+T cells, and M1 and M2-like macrophages was performed, respectively using Monocle 2 (version 2.8.0) (18). We then performed differential gene expression analysis using the differential Gene Test function to identify significant genes (BH-corrected P < 0.01). Cellular ordering of these genes was in an unsupervised manner. Trajectory construction was then performed after dimensionality reduction and cell sorting with default parameters.

### Calculation of Functional Module Scores

To evaluate the potential functionality of the cell cluster of interest, we calculated the scores of functional modules of the cell cluster, using the AddModuleScore function in Seurat. The gene lists for T cells signature and macrophages were listed in **Tables S1** and **S2**, respectively. The average expression levels of the corresponding cluster were subtracted by controlling the aggregated expression of the feature set. All analyzed genes were binned based on average expression, and control characteristics were randomly selected from each bin.

### SCENIC Analysis

Analysis of transcription factors (TF) was implemented using the Python package pySCENIC36 (version 0.9.19) (19) Co-expression modules were constructed by GRNBoost, and motif datasets (hg19-500bp-upstream-7species.mc9nr.feather,hg19-tss-centered-10kb-7species.mc9nr.feather) were used to construct regulons. The input data were normalized expression matrices from Seurat (R package, version 3.1.1).

### Cell-Cell Interaction Network Analysis

The ligand–receptor interactions among immune cells from the HSIL, tumor and lymph node metastatic were mapped using the CellPhoneDB algorithm (www.cellphonedb.org) (20). This method determined the potential cellular ligand–receptor interactions between the cell clusters based on the gene expression level. The significance of the cellular interactions was calculated based on the 1000 times permutation test. In the current study, we performed the cellular interactions for the ligands and receptors expressed in at least 25% of the cell subsets. We excluded the cellular interactions within the identical cell clusters, the interactions between the collagens and between the cell subsets account for less than 0.1% of the total cells. Those ligand–receptor interactions with p < 0.05 from the permutation tests was considered statistically significant.

### Correlation to Public Datasets

The TCGA-CESC data was used to evaluate the prognostic effect of individual genes or gene sets originating specific cell clusters. The patient cohorts was divided into high and low expression groups according to the median value of the normalized mean expression of strong marker genes (logFC > 2). When there was no significant difference in patients’ age, tumor stage and Grade between high and low groups, we performed further analysis. Kaplan–Meier survival curves and P values were generated by R package “survminer”.

### Statistical Analysis

Statistical analysis was performed using SPSS 20.0 (Chicago, IL, USA), and statistical significance was determined with a t test. The p values were calculated. Unless specifically stated, p < 0.05 was considered statistically significant.

## Result

### scRNA Sequencing Reveals the Unique Immune Landscape During CC Progression

**Figure 1:**
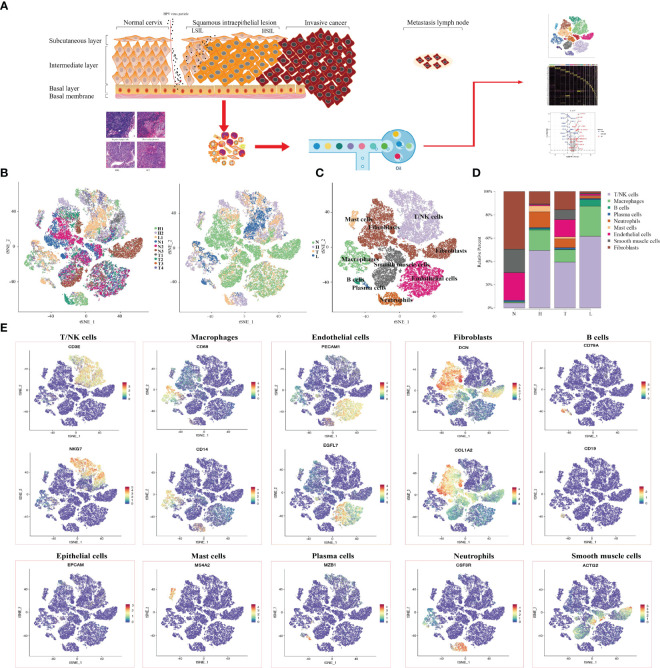
Single-Cell profiling of diverse immune cells from four groups (Normal cervix, HSIL, tumor and metastatic lymph nodes). Overview of the study workflow **(A)**. t-SNE plot and proportions of all cells annotated by the ten patients from four groups **(B)**. t-SNE plot of all cell types across samples from four groups **(C)**. Proportions of all cell types in each group **(D)**. Expression of cell-type-specific marker genes illustrated in t-SNE plots **(E)**.

In order to reveal the changing characteristics of immune microenvironment during CC progression, we obtained 10 × Genomics scRNA sequencing datasets from fresh samples, which spanned from normal cervix (n=3), HSIL (n=2), cervical tumors (n=4), and metastatic lymph nodes (n=1) (**Figure 1A**). Then, we used t-distributed stochastic neighbor embedding (t-SNE), an unsupervised nonlinear dimensionality reduction algorithm, to distinguish cell types according to the relative gene expression values. Overall, after initial quality control, a total of 59913 cells were retained and differentiated into 15 different clusters (clusters 1~15), which were visualized as a two-dimensional t-SNE map (**Figures S1A, B**). Of these, 30968, 10944, 11660 and 6341 cells were derived from normal cervix, HSIL, tumor and lymph node tissue (**Figure S1C**). By comparing the gene expression characteristics of epithelial/tumor cells, immune cells (T/NK, macrophages, neutrophils, mast cells and B cells) and mesenchymal cells (endothelial cells, fibroblasts and smooth muscle cells), these cell clusters were sorted into several main cell types (**Figures 1B, C, E**). The t-SNE plots showed the expression of cell type specific marker genes. These cell populations were unevenly distributed in patients or lesion sites (**Figure S1D**).

Then, we analyzed the changing trend of cell number in four groups (N, H, T and L) (**Figure 1D**). In normal tissues, most cell types were fibroblast (49.4%), endothelial cells (23.8%) and smooth muscle cells (20.3%), while the number of immune cells including (T/NK cells. Macrophages, B cells, plasma cells and Mast cells) were 5.2% and neutrophils were scarce. In HSIL tissue, the most types of immune cells were T/NK cells (49.4%), macrophages (17.6%) and neutrophils (13.5%), while the number of mesenchymal cells was decreasing. These results indicated that there is significant immune infiltration in the early stage of CC progression. Among the tumors, T/NK cells (39.4%), endothelial cells (14.9%) and fibroblasts (15.6%) were the most. However, the proportion of T/NK cells (61.6%) and macrophages (25.8%) in metastatic lymph nodes was higher, but the proportion of other types of cells was lower compared with other groups.

### Characterization of Single-Cell Expression Profiles for NK/T Cells Across Different Lesions

**Figure 2:**
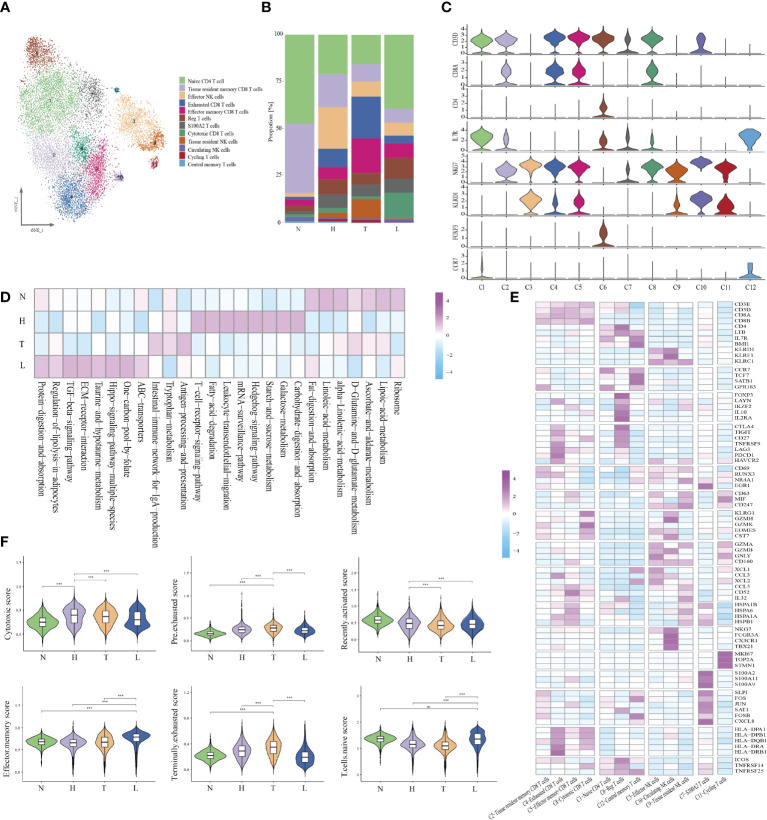
Identifying distinct NK/T cell clusters in all samples. t-SNE projection of 12 subsets of NK/T cells (each dot corresponds to one single cell) **(A)**. Proportions of all cell types clusters in each group **(B)**. Violin plots presenting the distribution module score for selected genes for each group **(C)**. Heatmap indicating the expression of selected gene sets in NK/T subtypes **(D)**. Heatmap indicating the enrichment of signaling pathway in four groups (GSVA KEGG) **(E)**. Violin plots showing the scores of functional modules for four groups, using the AddModuleScore function. Error bars indicated the means ± SD. (***P < 0.001). NS, no significant **(F)**.

Given the central role of NK/T cells in tumor immunity, we focused on the characterization of this cell type. We subclustered the transcriptomic profiles of 15229 NK/T cells. A total of 12 stable clusters emerged (**Figure 2A**). Four clusters (C2, C4, C5, and C8), showed high expression of CD8A and CD8B, defined as CD8+ T, three clusters (C1, C6, and C12) exhibited high expression level of CD4, and IL7R. defined as CD4+ T cells and three clusters (C3, C9, and C10) exhibited high expression of NKG7, and CD160, defined as NK cells. Cluster 7 with high expression of S100A family was defined as C7-S100A2 T cell, and cluster 11 with high expression of cycling-genes such as MKI67, TOP2A, and STMN1, was defined as cycling T cells. Then, we compared the number of CD8, CD4, and NK cells between different groups. The percentage of CD8 T cells in normal, HSIL, tumor and lymph node were 9.3%, 32.0%, 38.1%, and 20.6%, respectively. The percentages of CD4 T cells were 12.8%, 30.6%, 19.2%, and 38%, while the percentages of NK cells were 2.0%, 51.7%, 33.8% and 12.4% (**Figures 2B, C**). These results suggested that lesion in HSIL stage can activate the immune response. GSVA analysis showed high enrichment of T cell receptor signaling pathway, leukocyte transendothelial migration, B/NK/T cell activation in HSIL tissue. In tumor tissue, cells presented high enrichment of antigen processing and presentation, NK cell medicated cytotoxicity, and NK cell activation. In lymphoid tissue, cells were related to the enrichment of dendritic cell chemotaxis, mast cell activation and positive regulation of chemotaxis. In addition, these cells also exhibited high enrichment of ECM-receptor interaction, TGF-beta signaling pathway and ABC transporters (**Figure 2D** and **Figure S2**). We also found that high expression of immune-related genes in HSIL exhibited a protective function, while was associated with worse prognosis in tumor tissue (**Figure S3**).

Then, we analyzed each cluster with specific characteristics (**Figure 2E**). The cells of the first CD8 clusters (cluster 2) expressed resident marker genes such as CD69, EGR1 and RUNX3 and were defined as tissue-resident CD8 T. Cluster 4, exhibited high expression levels of exhaustion markers CTLA4, PDCD1, LAG3, and HAVCR2. Meanwhile, they also presented high expression of antigen presentation genes, such as HLA-DPA1, HLA-DPB1 and HLA-DRA, and was defined as exhausted CD8 T cells. Cluster 5 uniquely expressed a variety of heat-shock proteins (HSPA1B, HSPB1, and HSPA1A), and cytokines. (CD52, CCL5, and IL32), and was resemble with a group of dysfunctional CD8 T cells recently identified in melanoma, esophageal squamous cell cancer and liver cancer (21–23). It is reported that extracellular HSPs can trigger immune responses. Some studies have reported that GRP94, a member of HSP90, can selectively enhance the production of cytokines through the indirect action of antigen presenting cells (APCs) (24). Earlier studies have also shown that HSPs can carry tumor-derived peptides that may induce T-cell mediated immune response, and can stimulate NK cells in the absence of antigenic peptides (25). Recently, some studies have reported that these cells may be situated between effector function and exhausted function (26). Cluster 8 displayed high expression of effector genes (GZMK, GZMH, NKG7 and CST7), which were defined as cytotoxic T cells.

Cells in cluster 1 exhibited high expression of naive-related genes such as CCR7, TCF7, LEF1, and SEEL, which were defined as naive CD4 T cell. Cluster 6 highly expressed CD4, IL2RA, and FOXP3 and was defined as regulatory T cells. Cluster 12 showed high expression of LTB and IL7R, which was defined as central memory T cells. Then, we analyzed the specific characteristics of three NK clusters. Cluster 3 exhibited high expression of effector genes (GZMA, GZMB, GNLY and CD160), as well as chemokines (XCL1, XCL2 and CCL3), defined as effector NK cells. Cluster 10, had high expression of FGFBP2, CX3CR1 and FCGR3A, but low expression of resident genes. Due to the high expression of FCGR3A, these cells may be recruited from peripheral blood, suggesting that peripheral NKs are the main source of tumor-infiltrating NKs. Cluster 9 presented high expression of resident genes (NR4A1, RUNX3 and CD69) and secreted different cytokines, which were defined as tissue-resident NK cells. Meanwhile, cluster 10 cell highly expressed CD63, CD247 and MIF, indicating that these cells were related to the function of macrophages. Cluster 7 cells presented a high expression of the S100A family. The S100A family plays an important role in the formation and organization of immunological synapse between T cells and antigen-presenting cells, which are formed through intercellular binding and intracellular signaling transduction (27). Cluster 11 presented high expression of MKI67, TOP2A, and STMN1, indicating the enrichment of proliferative immune cells (**Figure 2E**).

In order to evaluate the potential function of each cell cluster and confirm the immune state of each stage during CC progression, we calculated the scores of functional modules for the cell cluster, using the AddModuleScore function. It is worth noting that cluster 4 exhibited higher pre-exhausted and terminally exhausted scores and cluster 2 exhibited higher effector memory score. Cluster 3, 5, 8, and 10 presented higher cytotoxic scores, while cluster 1 exhibited higher naive scores. Cluster 6 was associated with a high regulatory score and cluster 11 exhibited higher proliferative scores (**Figure S4**). According to the distribution of cell number, we found that HSIL tissue had high enrichment of cluster 2, 3, and 12, indicating the low activation of adaptive or innate immunity, which may be related to persistent HPV infection and abnormal cell infiltration. The tumor tissue exhibited high infiltration of cluster 4, 5, and 9, which was related to the state of immunosuppressive. However, metastatic lymph node was rich in naive and effective memory cells, which represented the early stage of immune activation (**Figure 2F**).

### Different Immune Status of NK/T Cells in HSIL, Tumor and Metastatic Lymph Node Tissue

**Figure 3:**
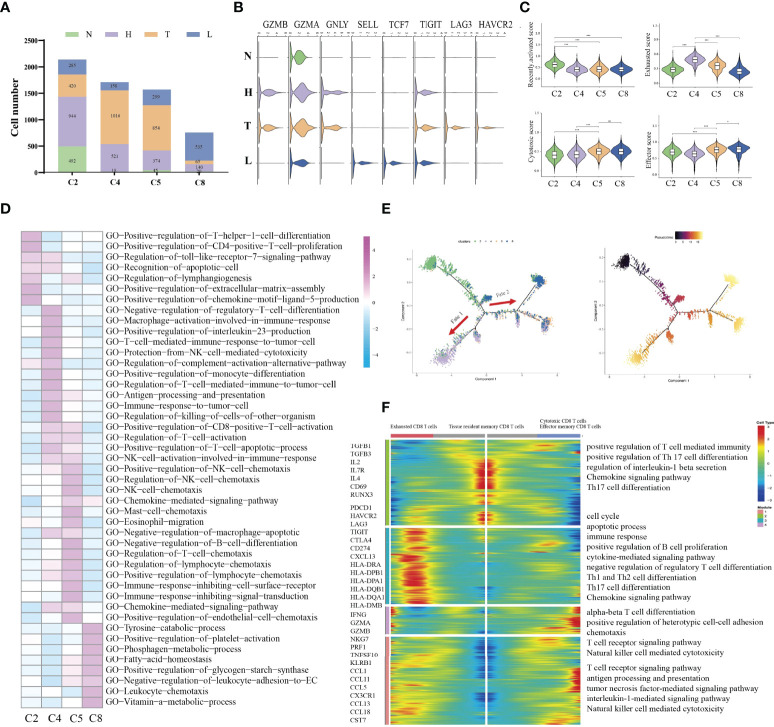
Difference of immune state among four groups (N, H, T, and L). Proportions of each CD8+ T cell cluster among four groups **(A)**. Violin plots showing the distribution module score for selected genes for each CD8+ T cells cluster. Error bars indicated the means ± SD **(B)**. Violin plots showing the scores of functional modules for each cell CD8+ T cluster, using the AddModuleScore function. (*P < 0.05; ***P < 0.001) **(C)**. Heatmap indicating the enrichment of signaling pathway among four CD8+ T cells cluster (GSVA GO) **(D)**. Trajectory of all CD8+ T cell clusters from all group along pseudotime in a two-dimensional state-space defined by Monocle2. Each point corresponds to a single cell, and each color represents a CD8+ T cell cluster **(E)**. Heatmap indicating the differentially expressed genes (rows) along the pseudotime **(F)**. NS, no significant.

Immune cell types exhibited different tissue preferences. We quantified tissue enrichment based on the cell numbers in each stage and explored the dynamic immune states and cell transitions in tumor-infiltrated CD8+ T cells. C2-tissue resident memory CD8 T cells, were mainly enriched in HSIL, and exhibited high expression level of IL2, IL7R, IL4 and CD69, which represented inactivate status of immune response (**Figures 3A, B**). Similarly, these cells showed higher effector memory, recently activation, and naive score compared to other groups (**Figure 3C** and **Figure S4**). Various studies have reported that tissue-resident memory CD8+T cells are an important first line of defense from infection in peripheral non-lymphoid tissues, such as the mucosal tissues of the respiratory, digestive, and urogenital tracts (28). This memory T cell subset is established late during resolution of primary infection of those tissues, has a distinct genetic signature (29). C4-exhausted CD8 T cells, were enriched in tumors, and exhibited high expression levels of immune checkpoint genes, antigen presentation and co-stimulatory factors. Meanwhile, these cells exhibited high exhausted score than other groups (**Figure 3C** and **Figure S4**). C5-effector memory CD8 T cells, were mainly derived from tumors, and exhibited high expression of CCL1, CCL11, NKG7, GZMB, PRF1, TNFSF10 and KLRB1. They also had high effector and cytotoxicity scores, indicating that effector memory CD8 T cell were the main effector cell type in tumors (**Figure 3C** and **Figure S4**). C8-cytotoxic CD8 T cells exhibited high expression of GZMA, and CX3CR1 as well as immature genes (CCR7, LEF1, SELL and TCF7) and had higher naive scores than other groups, suggesting the early stage of immune response (**Figure 3C** and **Figure S4**). Meanwhile, GSVA further identified the biological functional differences of NK/T cells among different clusters (**Figure 3D** and **Figure S5**).

Then, we performed pseudotime trajectory analysis using Monocle2 to order each CD8+ T cell along trajectories according to their expression and transition profiles (**Figure 3E**). C2 cells were at the beginning of the trajectory, and differentiated into two directions characterized by C4 cells, or C5/C8 cells, respectively. C4 (fate 1) represented an exhausted cell cluster. GSVA analysis showed high enrichment of immune activation signaling pathways, such as T cell-mediated immune response, positive regulation of killing of cells, protection of complement activation, and immune response to tumor cell (**Figure 3F**). Interestingly, about 43.1% of CD8 T cells in tumors were located in the branch. These outcomes indicated that CD8 T cells in tumors underwent an exhausted procedure. C5 and C8 cells represented another fate state (fate 2). C5 cells presented highly expressed chemotactic genes, which can attract NK cells, T cells and immature DCs. GSVA analysis showed high enrichment of immune cells-related signaling pathways, such as regulation of NK cell chemotaxis, mast cell chemotaxis, and T cells chemotaxis. Meanwhile, these cells were associated with NK cell activation, CD8 positive alpha-beta T cell activation and T cell apoptotic process. These outcomes indicated that these cells can regulate other immune cells function by secreting various cytokines. Thus, cells in cluster 5 can be considered as a transient state between effector function and exhausted function. Meanwhile, we found that about 36.3% of CD8 T cells in tumors were included in cluster 5. C8 presented a high expression of cytotoxic genes such as GZMH, GZMK and TNFSF10, but low expression of immune checkpoint genes and antigen-presentation genes. Meanwhile, it also presented high expression of naive genes. Interestingly, the majority of CD8 T cells in lymph node were located in the branch.

Overall, our study first demonstrated that HSIL was a low activated immune state (innate immunity), which is characterized by high infiltration of tissue resident memory CD8 T cells. Tumor presented an immunosuppressive state, which was characterized by infiltration of exhausted CD8 T cells. Metastatic lymph nodes had low activation of immune response, which was characterized by effector memory CD8 T cells and cytotoxic CD8 T cells, indicating early activate stage of immune response.

### Characterization of Myeloid Cells Across Different Lesions

**Figure 4:**
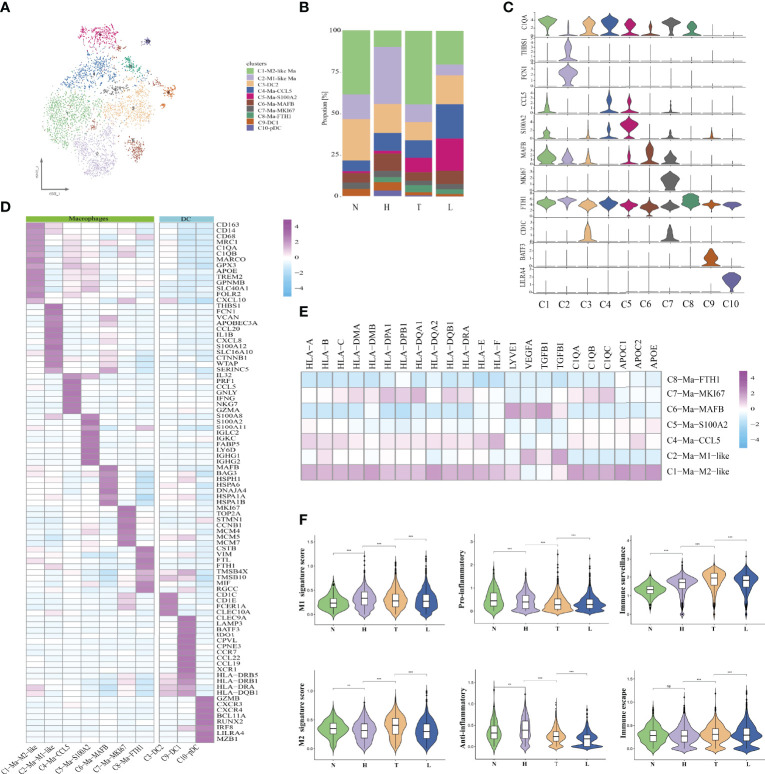
Identifying distinct myeloid cell clusters in all samples. t-SNE projection of 10 subsets of myeloid cells (each dot corresponds to one single cell) shown in different colors **(A)**. Proportions of all cell types clusters among four groups **(B)**. Violin plots representing the distribution module score for selected genes for each cluster. Error bars indicated the means ± SD **(C)**. Heatmap indicating the expression of selected gene sets in myeloid cell subtypes **(D)**. Heatmap indicating the differentially expressed genes in seven macrophages subtypes **(E)**. Violin plots showing the scores of functional modules for each macrophages cluster, using the AddModuleScore function. (**P < 0.01; ***P < 0.001) **(F)**. NS, no significant.

Next, we performed unsupervised clustering of all myeloid cells. A total of 10 clusters emerged within the myeloid lineage, including seven clusters (C1, C2, C4, C5, C6, C7, C8) of macrophages (CD163, MRC1 and CD14) and three clusters (C3, C9, C10) of DCs (CD1C, LILRA4 and CLEC10A) (**Figure 4A**). Then, we compared the number of DCs and macrophages in different lesions. The number of myeloid cells in normal tissue was less than that in other groups. Then, we found that HSIL presented a high percentage of macrophages (35.5%) and DCs (47.5%), suggesting the activation of immune response in HSIL phase. Notably, the percentage of macrophages (33%) and DCs (29.4%) in lymph node were also higher than that in tumors (**Figure 4B**). GSVA showed that HSIL was related to high enrichment of TNF signaling pathway, VEGF signaling pathway, T/B cell receptor signaling pathway and T cell cytokines production. In tumors, immune activation-related signaling pathway (innate and adaptive immunity) had been highly enriched, including NK cell medicated cytotoxicity, B/T/mast cell activation, toll-like receptor signaling pathway and antigen presentation. Lymph node exhibited high enrichment of Th17 cell commitment, IL5 production, Th1 cell cytokines production and ECM receptor interaction, suggesting immune reconstitution (**Figures S6A, B**).

Seven macrophage clusters were identified in our data (**Figures 4C, D**), of which cluster 1 had high expression levels of C1QA, MARCO, APOE, and CXCL10, and was enriched in tumors and lymph nodes, defined as C1-Ma-C1QA. Meanwhile, we found that the up-regulated genes (CD163 and CD68) in C1-Ma-C1QA, were similar to the signatures of TAM, M1 and M2 macrophages. The co-existence of M1 and M2 signatures indicated that TAMs was more complex than the classical M1/M2 model. Cluster 2 had high expression levels of THSB1, FCN1, VCAN and S100A12, which was defined as C2-Ma-THBS1. Meanwhile, we found that pro-inflammatory cytokines (NLRP3 and IL1B), were expressed in almost all macrophage subpopulations, and the C2-Ma-THBS1 was the highest, which was consistent with the role of the NLRP3 inflammasome in activating IL-1B and regulating the balance of intestinal environment. GSVA showed that C1 and C2 were the main cell type of macrophages that played an immunomodulatory role (**Figure S7A**). C6-Ma-MAFB intriguingly expressed a number of classical monocytic genes such as transcription factors KLF2/4 and NR4A1/2 as well as heat-shock proteins (HSPH1). These macrophages specifically expressed lymphatic vessel endothelial hyaluronan receptor 1 (LYVE1) and VEGFA, but the expression level of HLA-related genes was low (**Figure 4E**), which is similar to the recently reported LYVE1_high_MHCII_low_ tissue-resident macrophages. Most of these cells resided alongside blood vessels and played a critical role in restraining inflammation. C7-Ma-MKI67 highly expressed cell cycle-associated genes, including STMN1, TOP2A, and MKI67. These cells also specifically expressed the minichromosome maintenance (MCM) protein family genes, including MCM4, MCM5, and MCM7. It is reported that MCMs are mainly expressed in all cycling cells throughout the cell cycle, although they are lost in quiescent and differentiated cells (30). GSVA confirmed that C7 cells also played an important role in regulating immune activation (similar to C1). C5-Ma-S100A8, expressed cathepsin genes (CSTA, and CSTD) and calcium-binding proteins (S100A8, S100A2 and S100A6) in a tumor-specific manner, which were important for ECM remodeling. In fact, cathepsin-secreting macrophages have the characteristics of promoting tumor cell migration and invasion (31). Interestingly, most of C5 macrophages were derived from lymph nodes. In addition, we found that C5-Ma-S100A8 also exhibited specifically express the genes of plasma cell markers (IGHG1, IGHG2, IGHG3, IGHG4 and MZB1), indicating C5 cells and plasma cells play a synergistic role in regulating the TME in lymph node. C8-Ma-SPP1 expressed high levels of TMSB4X, TMSB10 and CSTB. It is reported that TMSB10 has recently been recognized as being an important player in the metastatic cascade including tumor angiogenesis, invasion, and metastasis in papillary thyroid carcinoma (32). CSTB can regulate the polarization of macrophages in the process of proliferation, migration, invasion and epithelial-mesenchymal transition (EMT) of HCC cells (33). C4-Ma-GZMA also expressed high level of cytotoxicity genes such as GNLY, FING, NKG7 and GZMA, indicating that these cells had cytotoxicity (**Figure 4D**). Macrophages were significantly enriched in HSIL, tumors and lymph node and displayed different proportions. We could not clearly distinguish M1 and M2 macrophages using known marker genes such as FCGR3A (M1) and CD163 (M2), as they were both expressed in those cells (**Figure 4D**), consistent with a previous report (26). However, by calculating M1 and M2 signatures and pro- and anti-inflammatory scores as well as immune surveillance and immune escape using related gene sets, we observed a dominant M2-like phenotype (thought to have a pro-cancer role) in tumor tissue, and a M1-like phenotype in HSIL (thought to have an anti-cancer role). Notably, macrophages in HSIL exhibited higher anti- and pro-inflammatory score compared with those in tumor, indicating the complexity of TME. Importantly, tumor tissue exhibited an immune surveillance and escape scores, which further explained the adverse effects of immunotherapy (**Figure 4F**).

Then, we defined the DCs subcluster (**Figures 4C, D**). Three DC subsets, including plasmacytoid DC (pDC), cDC2, and cDC1 cells, were characterized by high expression of HLA-DRs and low expression of CD14. These cells were further distinguished by specific expression of LILRA4/BCL11A, CD1C/FCER1A, and XCR1/BATF3, respectively. C9-DCs expressed the classical DC1 marker genes (XCR1 and CLEC9A), representing mature DCs (CCR7 and LAMP3). Compared with DC1 cells, C3-DC (CD1C and FCER1A), also had the unique signatures of DC2. C3-DC cells also showed a high expression of CD1E in the absence of CCR7, HLA-DRA, and CCL19. C10-DC did not correspond to any classical DC subset *in vivo*. They presented a high expression of CLEC4C, LILRA4, and LILRA5, namely plasmacytoid DC (pDC). Traditional DCs are classified into two subsets: cDC1 and cDC2. Their main function is to obtain tumor antigen, migrate to lymph nodes, prime to T cells (**Figure S7B**). In addition, we found that most of DC1, DC2 and pDC were from HSIL tissue or lymph node. cDC1 and cDC2 have been used as sources for vaccine immunotherapy and have shown encouraging immunological and clinical outcomes. Thus, the infiltrated DCs in HSIL or metastatic lesions may become the target of immunotherapy in the future.

### Difference of Macrophage Types Between HSIL and Tumor

**Figure 5:**
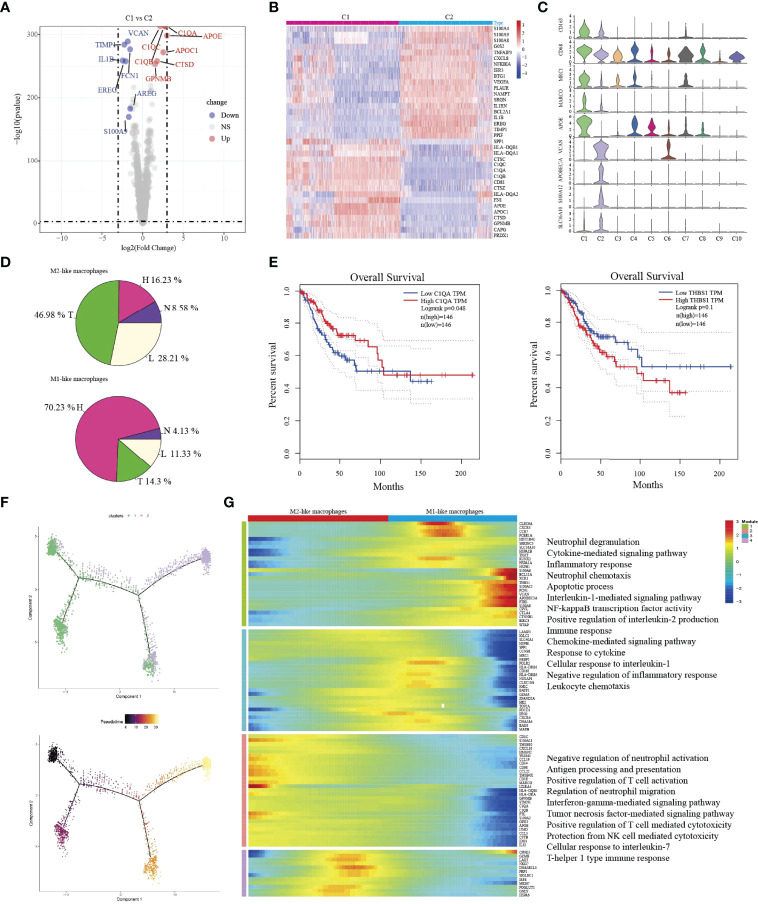
The transition of two types of macrophages during tumor progression. Volcano plot showing the top differently genes between two types of macrophages **(A)**. Heatmap indicating differently genes between two types of macrophages **(B)**. Violin plots showing the distribution module score for selected genes for each cluster. Error bars indicated the means ± SD **(C)**. Pie graph showing the proportions of cells among four groups **(D)**. Kaplan–Meier curve showing survival of C1QA, and THBS1 genes in TCGA-SCC patients **(E)**. Trajectory of both two macrophage clusters along pseudotime in a two-dimensional state-space defined by Monocle2. Each point corresponds to a single cell, and each color represents a macrophage cluster **(F)**. Heatmap indicating the differentially expressed genes (rows) along the pseudotime **(G)**.

C1-Ma-C1QA and C2-Ma-THBS1 macrophages are the main enriched cell types in tumor/lymph node and HSIL, respectively. Then, we compared the difference between the two macrophages. C1-Ma-C1QA highly expressed MRC1, APOC1, GPNMB and CTSD, while C2-Ma-THBS1 highly expressed VCAN, TIMP1, IL1B, EREG, and FCN1(**Figures 5A–C**). In addition, C1 cells also presented high expression of antigen-presentation genes such as HLA-DQB1, HLA-DQA1 and HLA-DQA2, indicating the subpopulation was related to immune activation. C2 cells were associated with adhesion molecular, such as S100A4, S100A9 and S100A8. GO analysis confirmed that C1 cells had high enrichment of immune effector process, neutrophil activation, cellular response to IF-gamma, and neutrophil activation (**Figure S8A**), while C2 cells were associated with lipopolysaccharide response, neutrophil migration, granulcyte chemotaxis and neutrophil chemotaxis (**Figure S8B**). Then, we found that C1 cells were the main macrophage type in tumor/lymph tissue, while C2 cells were the main type in HSIL (**Figure 5D**). Interestingly, high expression of C1QA had a better prognosis, but high expression of THBS1 was related to worse prognosis (**Figure 5E**). Then, we found that C2-Ma-THBS1 macrophages showed high score of M1 signature and anti-inflammatory score, while C1-Ma-C1QA had high score of M2 polarization signature and pro-inflammatory score (**Figures S9A–D**). Meanwhile, we also showed that C1-Ma-C1QA was associated with high immune surveillance and immune escape score (**Figures S9E, F**). Thus, C1-Ma-C1QA and C2-Ma-THBS1 also could be defined as M2-like macrophage and M1-like macrophage, respectively.

Then, we used Monocle2 for pseudotime trajectory analysis according to their expression and transition profiles. We observed C1-Ma-C1QA and C2-Ma-THBS1 cells were located at the two ends of trajectory and showed that C2-Ma-THBS1 and C1-Ma-C1QA formed a continuum, but had different expression features (**Figure 5F**). We observed that C2-Ma-THBS1 cells presented high enrichment of translation initiation, viral transcription, IL-1 signaling pathway, NK-kB signaling pathway, and cytokine-mediated signaling pathway. These results indicated that the activation of these signaling pathways imply a progression of tumor. C1-Ma-C1QA presented high enrichment of neutrophil activation, T cell activation, antigen processing and presentation and IF-gamma mediated signaling pathway (**Figure 5G**). In conclusion, these results suggested that when lesion progressed from HSIL to invasive tumor, the TME underwent a transition from pro-inflammatory (M1 signature) to anti-inflammatory (M2 signature).

### The Transcription Characteristics of B Cells Among Different Lesions

**Figure 6:**
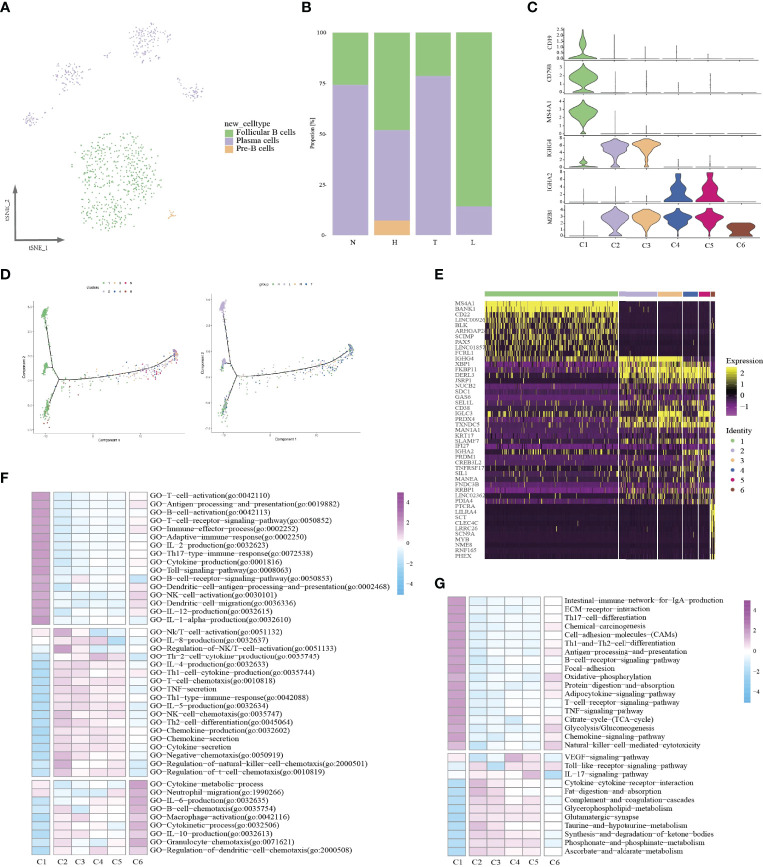
Identifying distinct B cell clusters in all samples. t-SNE projection of three cell types of B cells (each dot corresponds to one single cell) shown in different colors **(A)**. Proportions of all cell types clusters among four groups **(B)**. Violin plots representing the distribution module score for selected genes for each cluster. Error bars indicated the means ± SD **(C)**. Trajectory of all B cell clusters along pseudotime in a two-dimensional state-space defined by Monocle2. Each point corresponds to a single cell **(D)**. Heatmap indicating differently genes among six clusters **(E)**. Heatmap showing the enrichment of biological function in six B cell clusters (GSVA GO) **(F)**. Heatmap showing the enrichment of biological function in six B cell clusters (GSVA KEGG) **(G)**.

Some studies have shown that B cells have been a great impact on the immune regulation of TME and are closely related to the overall survival rate of tumor patients (34, 35). In this study, a total of 880 B cells were identified for further analysis. We re-clustered B cells and identified six sub-clusters (**Figures 6A, B**). Then, based on specific cell markers, we defined clusters 1 as follicular B cells (marked by MS4A1 and CD19), and cluster 2-5 as plasma B cells (marked by IGHG4 and MZB1) (**Figure 6C**). Low quality cluster (C6) will not be discussed further in this article. Notably, we found follicular B cells were mainly distributed in lymph node (357/518) and plasma B cells were distributed in tumor (**Figure 6B**). Cell reprogramming analysis at the single-cell level revealed the transition path of B cells from follicular B cells to plasma cells; this demonstrated the complex functions of B cells in tumor progression (**Figure 6D**). In addition, we also explored the two types of B cells, and their characteristics and functions during tumor progressive. Differential expression analysis revealed high expression of MS4A1, BANK1, CD22, BLK, ARHGAP24, SCIMP, PAX5 and FCRL1 in follicular B cells (**Figure 6E**). Plasma cells were related to high expression of IGHG4, XBP1, FKBP11, DERL3 and PRDX4. GSVA showed that follicular B cells were associated with immune cells activation and immune effector response. However, plasma cells were related to chemotaxis and cytokines production and Toll-like receptor signaling pathway (**Figures 6F, G**).

Then, we compared the specific functional characteristics of follicular B and plasma cells in different lesions. Notably, in HSIL tissue, follicular B cells exhibited high functional enrichment of chemical carcinogenesis, natural killer cell-medicated cytotocity and B cell receptor signaling pathway (**Figure S10A**). Meanwhile, we found that follicular B also exhibited specific functional enrichment in tumors, including TNF-signaling pathway, IL17 signaling pathway and toll-like signaling pathway, which demonstrated the activation of the natural immune system. Importantly, follicular B, was mainly enriched in lymph node, and exhibited chemokine signaling, T cell receptor signaling pathway and PPAR signaling pathway and antigen presentation, which can activate adapt immune. These results indicated that follicular B mainly came from lymph node. Similarly, plasma cells among different lesions had distinct expression programs (**Figure S10B**). In normal tissue or lymph node, plasma B presented high enrichment of IL-17 signaling pathway, B cell receptor signaling pathway, TNF signaling pathway, and antigen presentation. However, in HSIL or tumors, plasma B showed notable complement and coagulation cascades, chemical carcinogenesis and chemokine-cytokine receptor interaction.

### Complex Intercellular Communication Networks in HSIL, Tumor and Lymph Node

**Figure 7:**
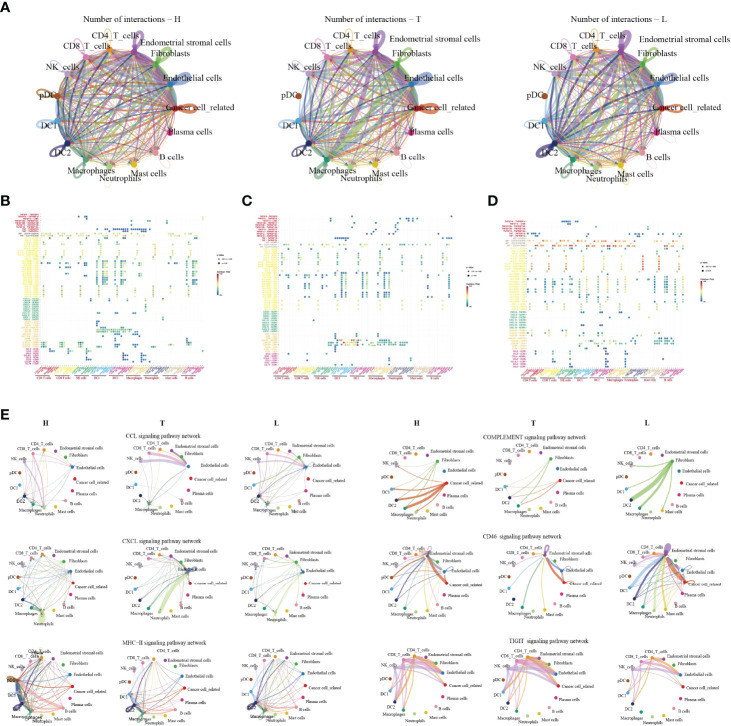
The dense network and multiple cellular connections. Circos plot showing the interactions between ligands and receptors across cell types in HSIL(left), tumor (middle), and lymph node (right) **(A)**. Bubble diagram showing MHC moleculars, immune receptors, chemokine and immune checkpint among different immune cells in HSIL **(B)**, tumor **(C)** and lymph node **(D)**. The association of different signaling pathways among different immune cells in HSIL, tumor and lymph node group **(E)**.

In order to systematically elucidate the complex cellular responses, we attempted to map the ligand-receptor communication network to better understand the cellular behaviour and the response to neighbouring cells in HSIL, tumor and lymph node, respectively. We considered the expression levels of ligands and receptors in each cell type and predicted the molecular interactions between cell populations through specific protein complexes. We then generated a potential intercellular communication networks among all cells in the three groups (**Figure 7A**).

Then, we compared the interactions of MHC molecules, chemokines, immune receptors, immunostimulators and immune checkpoints among three groups (**Figures 7B–D**). The results showed that MHC molecules, MIF-CD74/CXCR4, and MIF-CD74/CD44 in HSIL and lymph nodes were higher than those in tumor tissue. However, tumor tissue exhibited higher levels of immune checkpoint-related ligand-receptors, such as LGALS9-HAVCR2, LAGALS9-CD44, ICOSL-ICOS, ICOSL-CTLA4, and ICOSL-CD28. These results further proved the existence of immunosuppressive TME of tumor tissue. Then, we found that CLEC2D/C/E-KLRB1 exhibited strong communication among CD4 T cells, CD8 T cells and NK cells in HSIL, indicating potential immune activation. Last, we compared the differences between various signaling pathways (**Figure 7E**). The results showed that HSIL and lymph node tissue had strong communication in MHC, complement activation, and CD46 signaling pathways, which were related to immune regulation and activation. However, tumor tissue exhibited strong communication in CCLs, CXCLs, and TIGIT signaling pathways. These data further demonstrated different immune states in the process of CC development.

## Discussion

**Figure 8:**
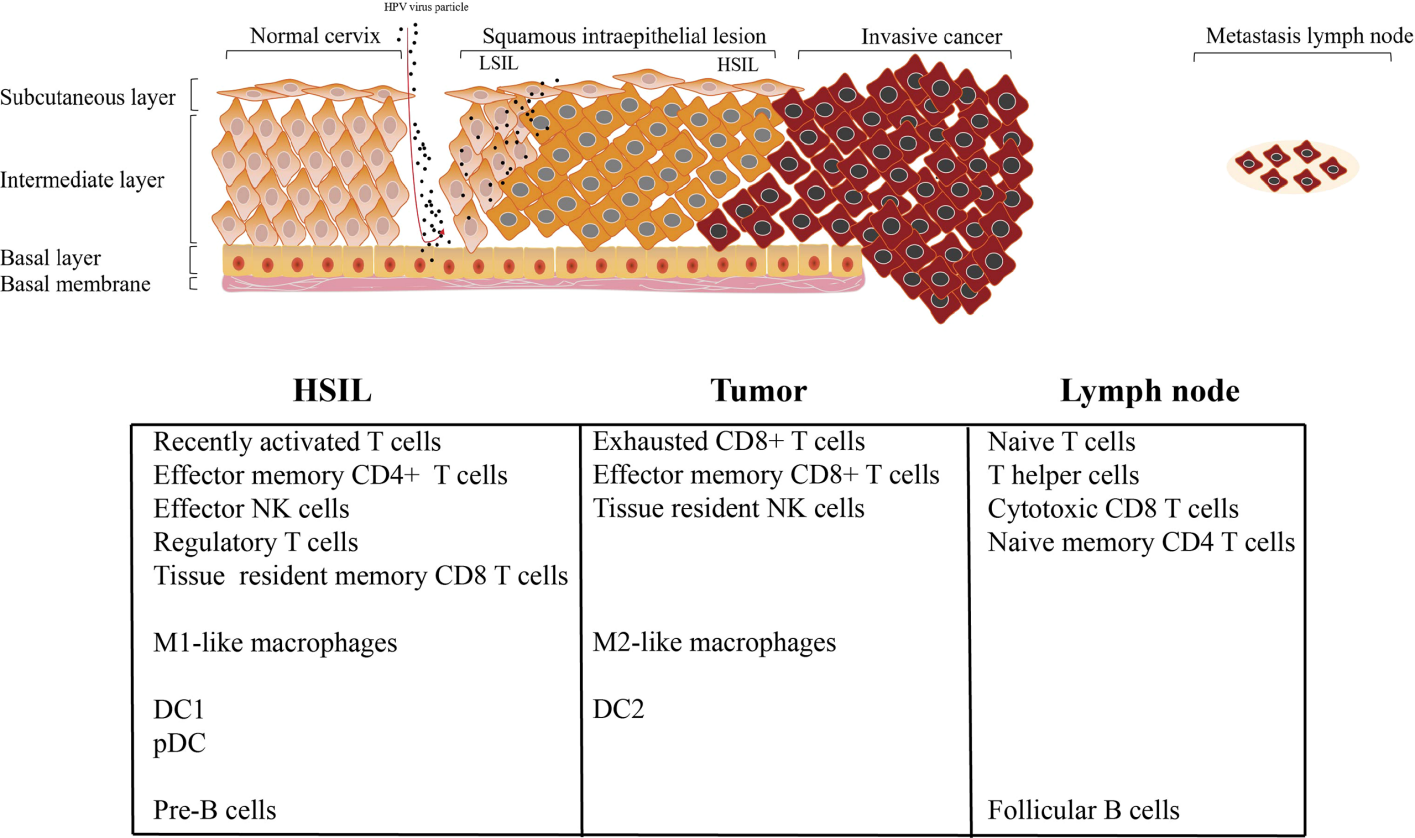
Summary of conclusion.

Recently, CC is considered as a preventable malignant disease, due to the clear etiology of HPV infection and the application of HPV vaccine (3, 4) With the continuous infection of HPV, various oncogenic genome, transcriptome, and epigenetic changes accumulate in epithelial cells in a gradual manner, affecting various signaling pathways driving CC (3, 4). TME targeting strategy provides a novel treatment option for cancer treatment (36); however, because the immune microenvironment of CC is largely unknown, these protocols have yet been widely applied in CC patients. For example, how HPV infection induces the occurrence of HSIL, why HSIL can progress to invasive tumor, and why cervical cancer occurs easily lymph node metastasis. No studies have addressed these questions of how TME affects the cancer cell during this process. Across multiple studies conducted over the years, including a tentative exploration of chromatin accessibility during HPV-derive CC, the best description for understanding CC transcriptome diversity at the single-cell level has not been determined. In this study, we first depicted the single-cell transcriptomic profiling of normal cervix, premalignant lesion (HSIL), primary cervical tumors, and metastatic lymph node, to reveal the dynamic change of TME subpopulations. Our results showed that there are significant differences in the infiltration level of immune cells at different stages of tumor progression. We then found that HSIL exhibited a low, recently activated TME, characterized by an infiltration of tissue resident CD8 T cell, effector NK cells, Treg, DC1, pDC, and M1-like macrophages. Tumor tissue showed a notable immunosuppressive TME with high infiltration level of exhausted CD8 T cells, resident NK cells and M2-like macrophages. Interestingly, early immune response were observed in metastatic lymph node, characterized by a high infiltration of CD4 T cells (naive T cell, and central memory T cell), cytotoxic CD8+ T cells, circling NK cells and effector memory CD8 T cells (**Figure 8**). These results deepen our current understanding of CC development and progression, and provide a novel therapeutic model for CC in the future.

Cervical HSIL is a precursor of HPV-associated CC and the basis of cancer progression, which has not been fully determined. Growing evidence indicates that the impaired local, rather than systemic immune functions plays a key role in the occurrence of cervical carcinogenesis (37). We found a low, recently activated TME in HSIL. Dendritic cells are the most effective antigen-presenting cells, which have the unique effect of initiating naive T cells and inducing their functional polarization. Importantly, HSIL tissue exhibited high enrichment of DC1 and pDC. It is reported that cDC1 excelled in the activation of CTL, which is a critical effector cell type of anti-tumor immunity. Regulatory T cells (CD4+ FOXP3+) are an essential part of balancing the effects of the immune system, so they are very important for immune homeostasis. They can suppress immune responses by fighting various infections and abnormal cells (38). The local accumulation of activated Tregs in vitally infected tissues has been shown to support immune evasion, resulting in the persistence of infection (39). Meanwhile, we found HSIL exhibited high infiltration of naive CD4+ and tissue resident CD8+ T cells. These cells were not anergic, but were inhibited by infiltrated Tregs by secreting IL-10 and TGF-b1. Thus, we speculated that HSIL tissue constructed an immune privilege, which is related to persistent HPV infection and lesion progression. Similar to lymphocytes, multiple myeloid cell subsets were found in the TME of HSIL, and pro-inflammatory and anti-tumor macrophages (M1-like macrophages) were the dominant cell subpopulations, which were also suppressed by Tregs. In fact, the outcome of lesion regression or progression will depend on which of two forces dominate the dialogue.

Different from immune microenvironment in HSIL tissue, tumor tissue exhibited a notable immunosuppression with high infiltration of exhausted CD8 T cells, resident NK cells and M2-like macrophages, which was consistent with other studies of lung cancer and liver cancer (40, 41). CD8+ T cells are the most important immune cells that regulate the tumor microenvironment (42). We observed the continuous evolution of CD8+ T cells in CC at different stages. Unlike the high infiltration of tissue-resident CD8+ T cell, tumors exhibited the most high infiltration of exhausted CD8+ T cells, which represented a dysfunction. Similar to our results, recent studies by Gu et al. reported that tumor tissue exhibited high enrichment of CD8 T cells with high expression level of immune checkpoint genes such as LAG3, PDCD1 and HAVCR2/TIM3 in CC tissue (43). In this study, we found that these cells also exerted indirect cytotoxicity by secreting chemotaxis, such as CCL1, CCL5, CLL11 and CX3CR1, which can attract NK cells, T cells and immature DCs to TME lesion. This is partly due to the chronic stimulation of tumor antigens and the continuous remodeling of TME, which may eventually alter the phenotype and function of immune subsets. Similar to previous studies, tumor tissue had more infiltration of M2-like macrophages, and it has anti-inflammatory and tumor promoting effects, which will further dampen the anti-tumor ability of cytotoxic CD8+ T cells (44, 45). In this study, we found that macrophages enriched in tumor tissue exhibited high expression of CD163 and CD68 (M2-like macrophages). It is reported that the intraepithelial filtration of CD68 + and CD163+ macrophages will increase, as the disease evolves from HPV infection and CIN to invasive disease (46). In addition, these cells contribute to the immune escape of tumors, and are thus related to tumor progression (46). A recent study found that CC expressing higher CCL22, and Foxp3 markers was more aggressive, resulting in poor overall survival, regardless of FIGO stage or disease subtypes (47). Further, the study also showed that the expression of CCL22 was mainly localized majorly to M2-like macrophages carrying CD163 positive (47). Meanwhile, these macrophages have regulatory, and can promote Tregs migration and differentiation.

Interestingly, metastatic lymph node exhibited the early stage of immune response, characterized by the infiltration of naive CD4 T cell, central memory CD4 T cell, circling NK cells, cytotoxic CD8+ T cells and effector memory CD8 T cells. In addition, macrophages with high expression of IGHG1 and IGHG2 (cluster C5-Ma-S100A8) were enriched in lymph node. Notably, the early activation marker of immune response CD25 was highly expressed in lymph nodes. Cytotoxic CD8+ T cells are a type of immune cell type that can kill abnormal cells. In lymph node, cluster 8 was the main CD8+ T cell type, and showed high expression of GZMK and KLRG1, which appeared to be distinct from conventional T cell subtype. Compared with conventional effector T cells, these cells expressed lower levels of cytotoxic markers (GZMB, GZMB, and GNLY) and high levels of naive markers (CCR7 and LEF1), and thus may possibly represent cells in a transition state from naive to effector T cells. Meanwhile, we also found that naive CD4 T cells were mainly cell types in metastatic lymph node, indicating that these cells were the source of cytotoxic CD8+ T cells. The adaptive immune response of effector memory CD8+ T cells (Tem) is considered to be a central component of the immune response to disseminated disease (48). However, despite the significant enhancement of effector memory CD8 T cells infiltration, the tumor developed, indicating that these T cells could not prevent the spread of CC.

Different immune microenvironment means different immunotherapy strategies. When the lesion develops to the HSIL stage, the tumor environment showed a low, and recently activated state. It is well known that cytokines can be used by tumors to suppress the immune response, or the immune system to induce immune response (49). Therefore, the addition of exogenous cytokines is conducive to the clarity of the abnormal cells or HPV infection. Among a variety of cytokines, ILs and IFNs have been the most widely and efficiently used in cancer treatment (50). In addition, we found that both HSIL and lymph nodes presented high infiltration of DCs. DCs vaccine is composed of powerful antigen-presenting cells (APCs) (51), which can induce effective immune responses, and may be a potential method for the treatment of HSIL or metastatic CC. Tumor-associated macrophages (TAMs), are an important component of tumor microenvironment in tumor tissue, which can regulate immune response and promote tumor progression (52). The researchers also pointed out that macrophage colony-stimulating factor (M-CSF) is vital for the shift of microglia/macrophage to M2 subtype, and induces tumor proliferation (51). BLZ945, a CSF inhibitor, has been tested to target TAMs with satisfactory survival with elimination of tumor cells and decrease of M2 in TAM (53). Thus, therapies targeting the transformation of macrophage may be effect to inhibit lesion from HSIL to tumor. Immunotherapy with immune checkpoint inhibitors (ICIs) has changed the treatment paradigm for various cancer types and improved outcomes for patients with advanced or metastatic cancer (54). In this study, we found that some CD8 T cells expressed high levels of immune checkpoint genes in tumor or metastatic lymph node, such as PD-1, PD-L1 and CTLA4, suggesting that ICIs can be effective for CC treatment. However, our study also had some limitations, such as the relatively low coverage of 3’ end sequencing and limited sample size. Furthermore, similar to most single-cell studies of observational nature, this hypothesis should be tested with more research. Regarding the cellular changes found in this study, RNA *in situ* hybridization and immunohistochemistry can help identify changes in specific cell subsets. Biological functional validations, such as cytokine measurements or cytotoxicity assays, was awaited to explore the underlying mechanisms. Therefor, the results should be interpreted with caution. Nevertheless, unlike published studies (43, 55), to the best of our knowledge, this study first provided an important understanding of TME at different stages of CC progression.

In conclusion, we analyzed the single-cell atlas of normal cervix, premalignant lesion (HSIL), primary cervical tumors, and metastatic lymph node. Our results elucidated the characteristic of TME changes from precancerous lesions to invasive tumor, to metastatic lymph node. These findings provide in-depth insights into the characteristics of CC and potential therapeutic methods in the future.

## Data Availability Statement

The datasets presented in this study can be found in online repositories. The names of the repository/repositories and accession number(s) can be found below: www.ebi.ac.uk/biostudies/arrayexpress/studies/. accession: E-MTAB-12305.

## Ethics Statement

This study was approved by the institutional ethics review board of Shanghai OB/GYN Hospital.

## Author Contributions

CL contributed equally to this work. CL collected the data and did the statistical analysis, CL and KH organized and submitted the manuscript. KH guided the whole process. All authors contributed to the article and approved the submitted version.

## Funding

This study was supported by the National Natural Science Foundation of China (Grant No. 82173188) to KH, the Clinical Research Plan of SHDC (SHDC2020CR1045B, SHDC2020CR6009) to KH, the Shanghai Municipal Health Commission (20194Y0085) to CL, and the Shanghai “Rising Stars of Medical Talent” Youth Development Program (SHWSRS2020087) to CL.

## Conflict of Interest

The authors declare that the research was conducted in the absence of any commercial or financial relationships that could be construed as a potential conflict of interest.

## Publisher’s Note

All claims expressed in this article are solely those of the authors and do not necessarily represent those of their affiliated organizations, or those of the publisher, the editors and the reviewers. Any product that may be evaluated in this article, or claim that may be made by its manufacturer, is not guaranteed or endorsed by the publisher.

## Supplementary Material

Supplementary Figure 1 The distribution of cells in different groups. t-SNE projection of 15 cluster of all myeloid cells (each dot corresponds to one single cell) **(A)**. t-SNE showing the distribution of cells in four groups **(B)**. The proportions of all cells annotated in each group **(C)**. t-SNE plot of all cell types annotated in each groups **(D)**.

Supplementary Figure 2 The functional enrichment of NK/T cells among different groups (N, H, T, and L).

Supplementary Figure 3 Violin plots showing the scores of functional modules for each cell cluster, using the AddModuleScore function.

Supplementary Figure 4 Kaplan–Meier curve showing survival of selected immune-related genes expressed in H **(A)** and T samples **(B)**. All patients were from TCGA database.

Supplementary Figure 5 The difference of functional enrichment among different clusters (C2, 4, 5, and C8).

Supplementary Figure 6 Biological difference of all myeloid cells among four groups (N, H, T, and L). Heatmap showing the enrichment of biological function in four groups (KEGG) **(A)**. Heatmap showing the enrichment of biological function in four groups (GO) **(B)**.

Supplementary Figure 7 The function of macrophages and DCs among different clusters. Heatmap showing the enrichment of biological function (GO) and (KEGG) among seven macrophage clusters **(A)**. Heatmap showing the enrichment of biological function in three DC clusters (GO) an (KEGG) among three DCs clusters **(B)**.

Supplementary Figure 8 The functional difference between C1-Ma-C1QA and C2-Ma-THBS1 macrophages. Circle map showing the GO biological function with corresponding genes in C1-Ma-C1QA macrophage **(A)**. Circle map showing the GO biological function with corresponding genes in C2-Ma-THBS1 macrophage **(B)**.

Supplementary Figure 9 Comparison of immune function between C1-Ma-C1QA and C2-Ma-THBS1 macrophages. Violin plots showing the AddModuleScore differences of M1-polarization **(A)**, M2-polarization **(B)**, pro-inflammatory **(C)**, anti-inflammatory **(D)**, immune surveillance **(E)**, and immune escape **(F)** between C1-Ma-C1QA and C2-Ma-THBS1 macrophages.

Supplementary Figure 10 The function of follicular B cells and plasma cells among four groups (N, H, T and L). Heatmap showing the enrichment of biological function in follicular B cells among four groups **(A)**. Heatmap showing the enrichment of biological function in plasma cell among four groups **(B)**.
