# Corrigendum: Dissecting the single-cell transcriptome network of immune environment underlying cervical premalignant lesion, cervical cancer and metastatic lymph nodes

**DOI:** 10.3389/fimmu.2024.1386072

**Published:** 2024-02-23

**Authors:** Chunbo Li, Keqin Hua

**Affiliations:** ^1^ Department of Obstetrics and Gynecology, Obstetrics and Gynecology Hospital of Fudan University, Shanghai, China; ^2^ Shanghai Key Laboratory of Female Reproductive Endocrine Related Diseases, Shanghai, China

**Keywords:** cervical cancer, single-cell sequencing, tumor microenvironment, squamous cell carcinoma, immune cell

In the published article, there was an error in the **Data Availability** statement. The repository information and accession number were incorrect and should be updated. The correct **Data Availability** statement appears below.

**Data Availability Statement**

“The datasets presented in this study can be found in online repositories. The names of the repository/repositories and accession number(s) can be found below: www.ebi.ac.uk/biostudies/arrayexpress/studies/. accession: E-MTAB-12305.”

The authors apologize for this error and state that this does not change the scientific conclusions of the article in any way. The original article has been updated.

