# Supplementary figures and images for: Dissecting the Single-Cell Transcriptome Network of Immune Environment Underlying Cervical Premalignant Lesion, Cervical Cancer and Metastatic Lymph Nodes
Source: Front Immunol. 2022 Jun 24;13:897366. doi: 10.3389/fimmu.2022.897366 (PMC9263187; doi:10.3389/fimmu.2022.897366)

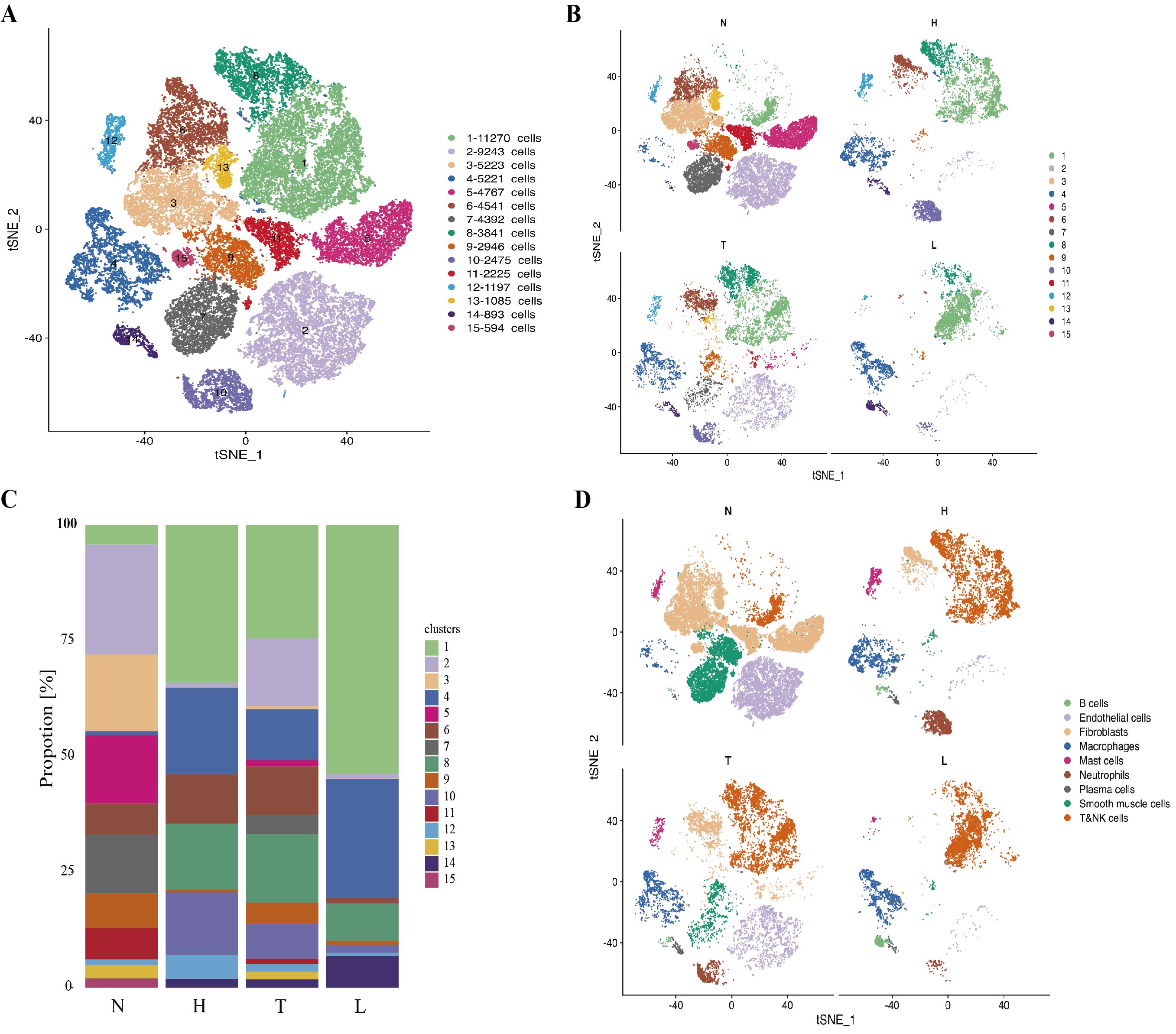

Supplement: Supplementary Figure 1 — The distribution of cells in different groups. t-SNE projection of 15 cluster of all myeloid cells (each dot corresponds to one single cell) (A). t-SNE showing the distribution of cells in four groups (B). The proportions of all cells annotated in each group (C). t-SNE plot of all cell types annotated in each groups (D). [file Image_1.jpeg]

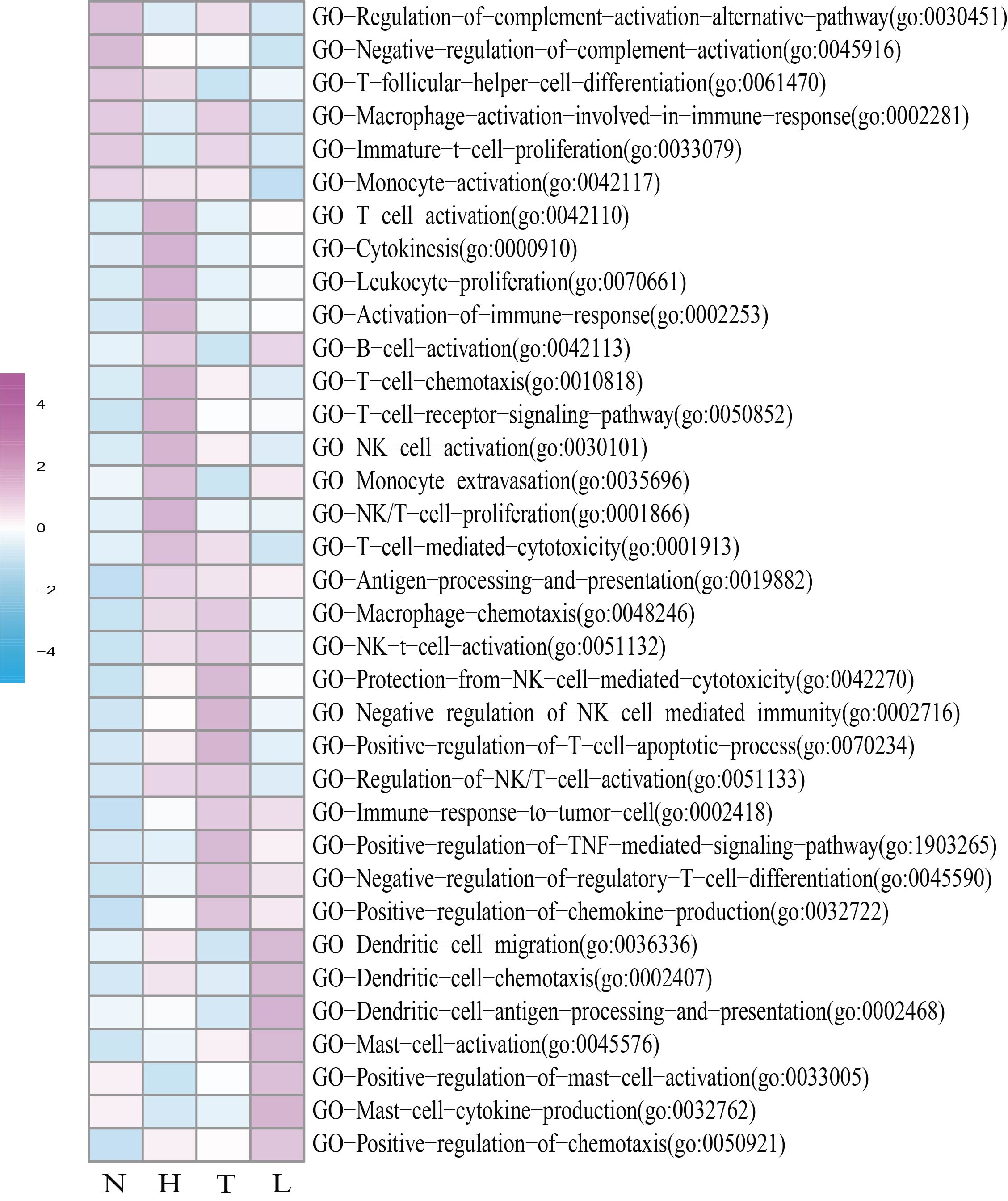

Supplement: Supplementary Figure 2 — The functional enrichment of NK/T cells among different groups (N, H, T, and L). [file Image_2.jpeg]

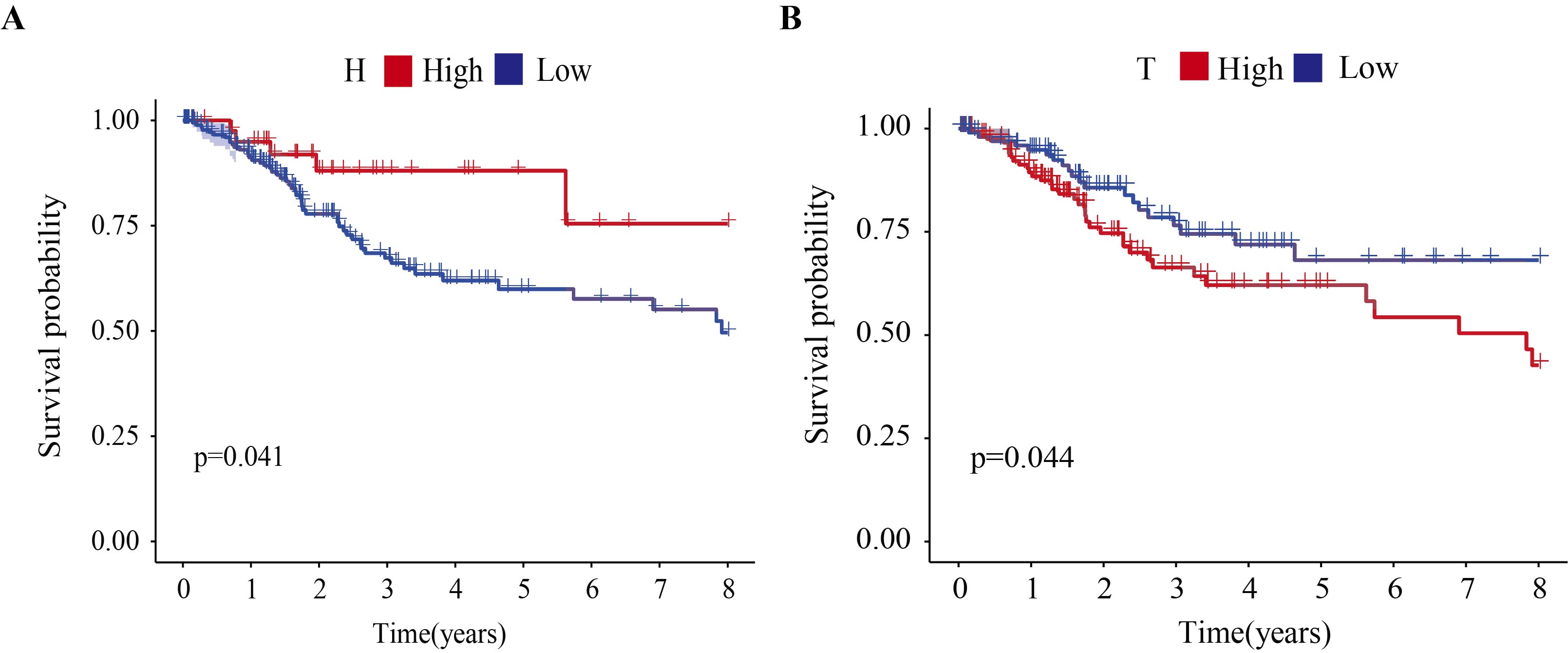

Supplement: Supplementary Figure 3 — Violin plots showing the scores of functional modules for each cell cluster, using the AddModuleScore function. [file Image_3.jpeg]

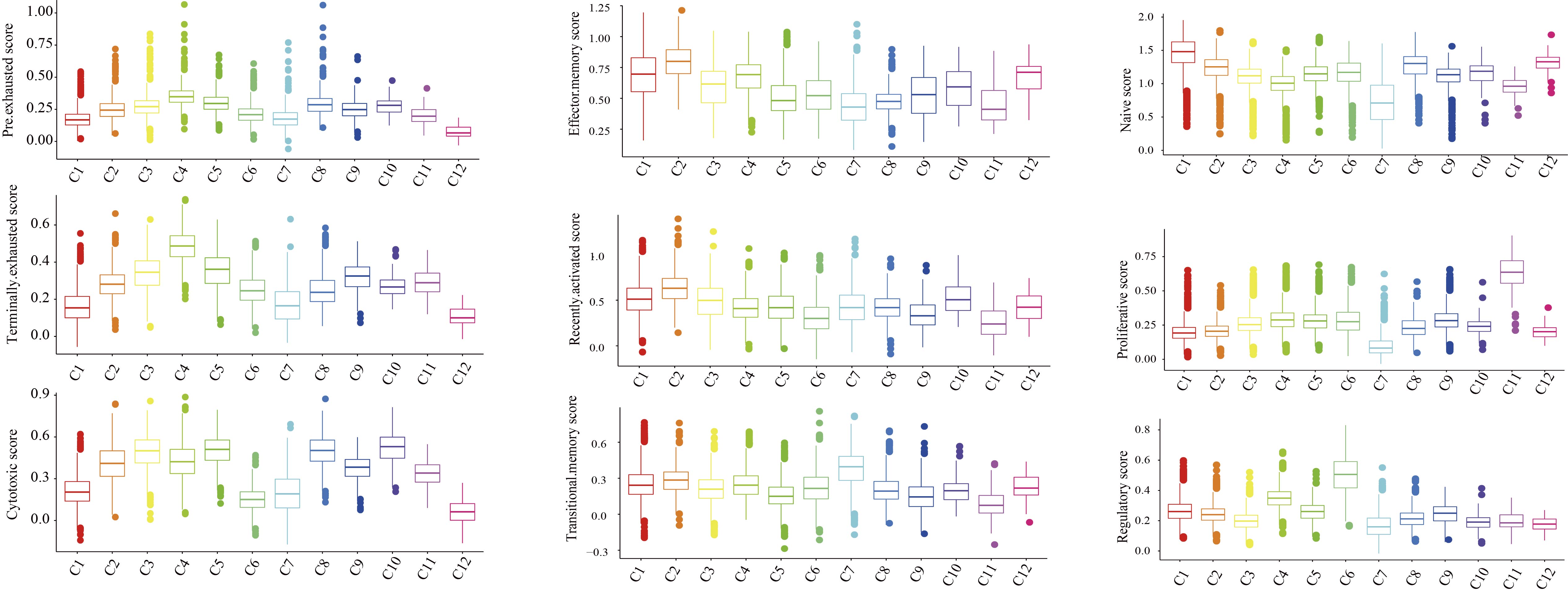

Supplement: Supplementary Figure 4 — Kaplan–Meier curve showing survival of selected immune-related genes expressed in H (A) and T samples (B). All patients were from TCGA database. [file Image_4.jpeg]

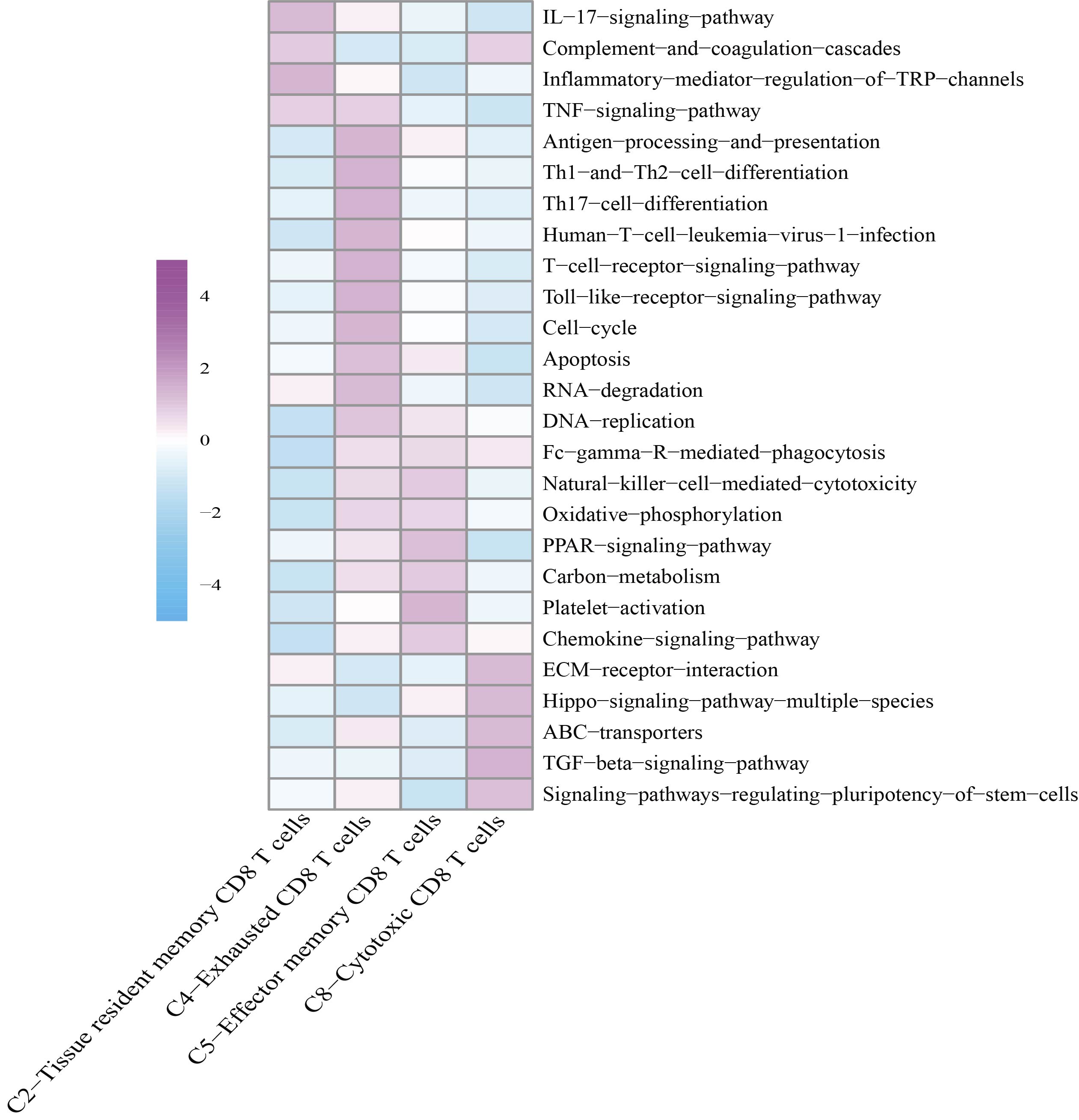

Supplement: Supplementary Figure 5 — The difference of functional enrichment among different clusters (C2, 4, 5, and C8). [file Image_5.jpeg]

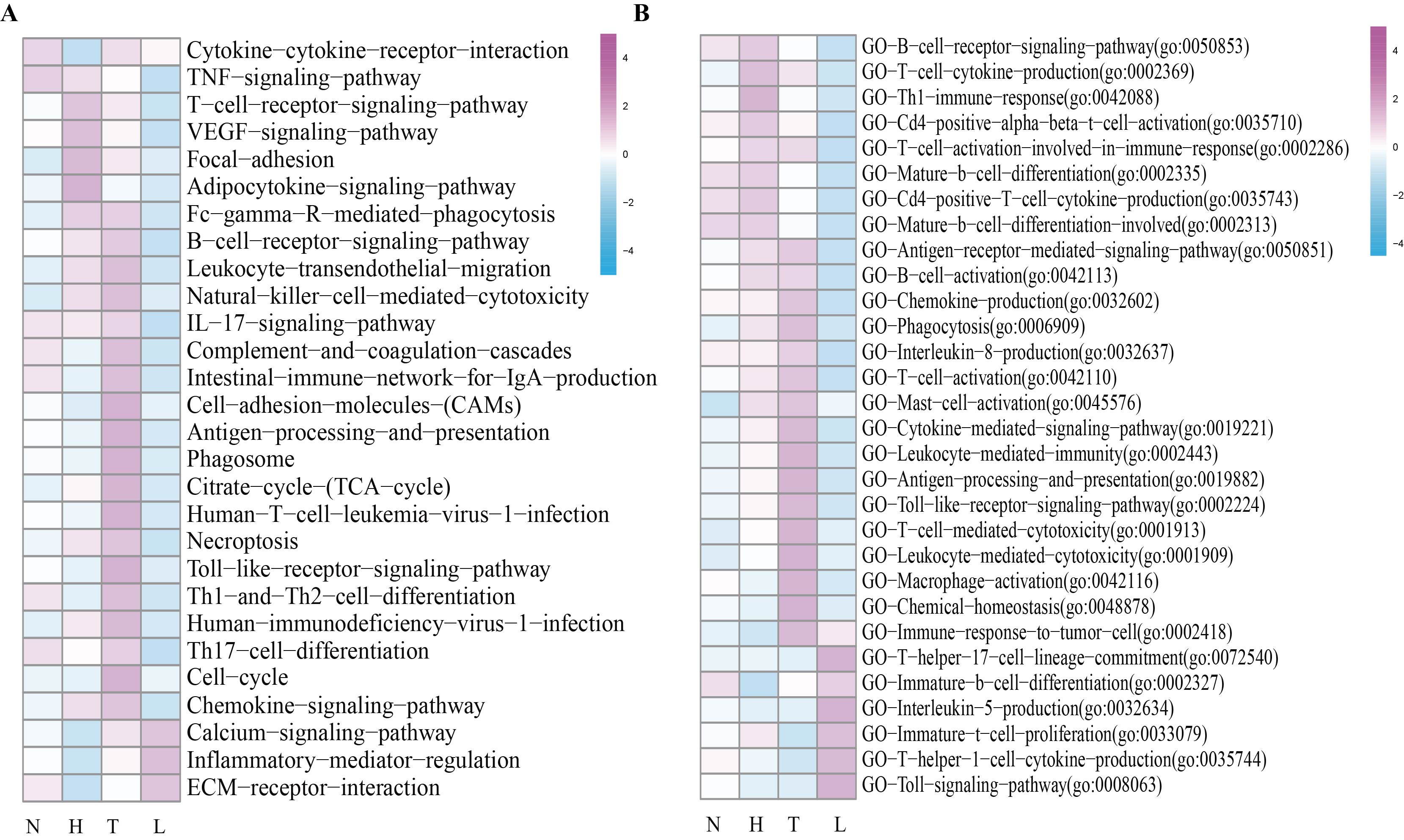

Supplement: Supplementary Figure 6 — Biological difference of all myeloid cells among four groups (N, H, T, and L). Heatmap showing the enrichment of biological function in four groups (KEGG) (A). Heatmap showing the enrichment of biological function in four groups (GO) (B). [file Image_6.jpeg]

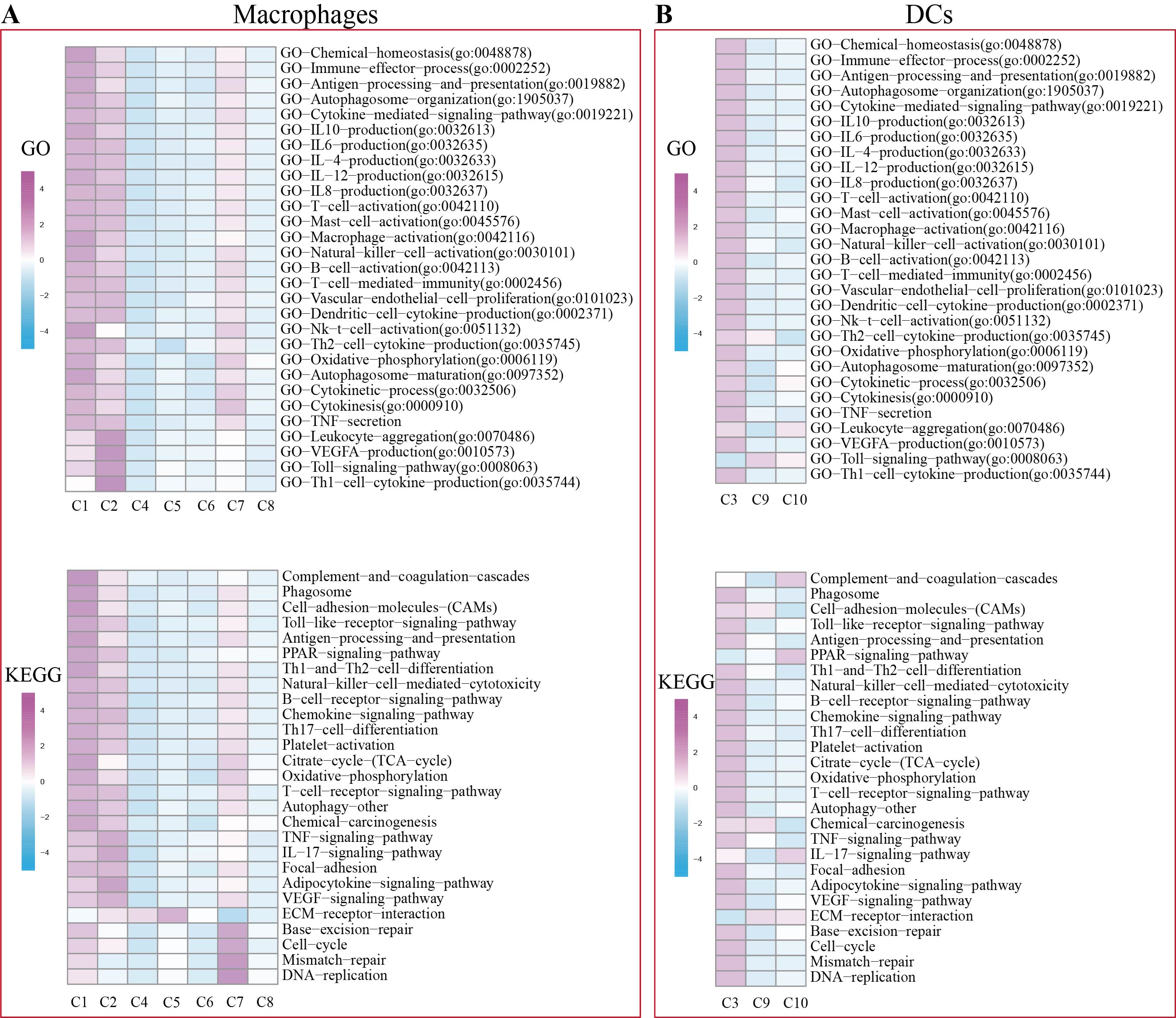

Supplement: Supplementary Figure 7 — The function of macrophages and DCs among different clusters. Heatmap showing the enrichment of biological function (GO) and (KEGG) among seven macrophage clusters (A). Heatmap showing the enrichment of biological function in three DC clusters (GO) an (KEGG) among three DCs clusters (B). [file Image_7.jpeg]

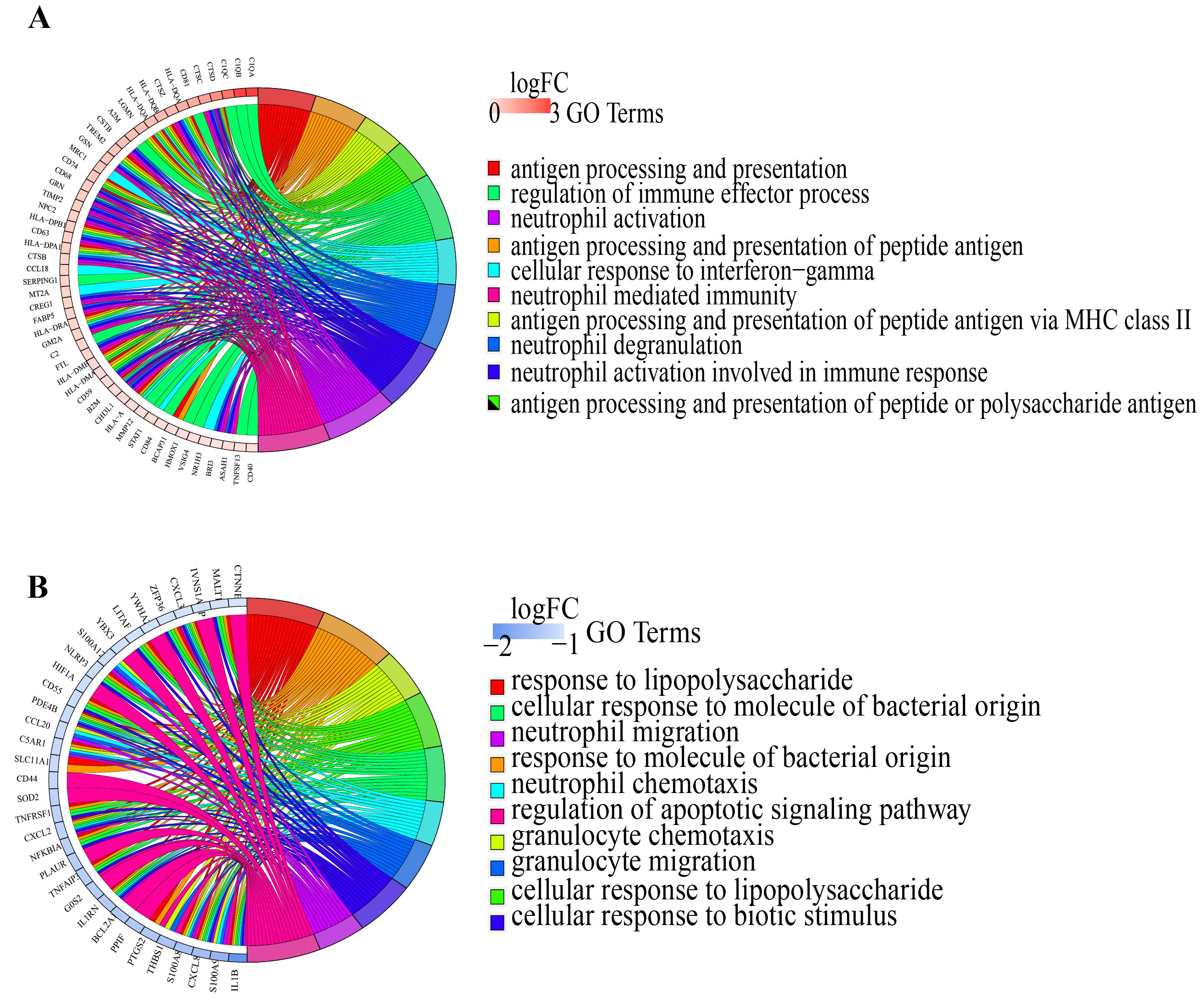

Supplement: Supplementary Figure 8 — The functional difference between C1-Ma-C1QA and C2-Ma-THBS1 macrophages. Circle map showing the GO biological function with corresponding genes in C1-Ma-C1QA macrophage (A). Circle map showing the GO biological function with corresponding genes in C2-Ma-THBS1 macrophage (B). [file Image_8.jpeg]

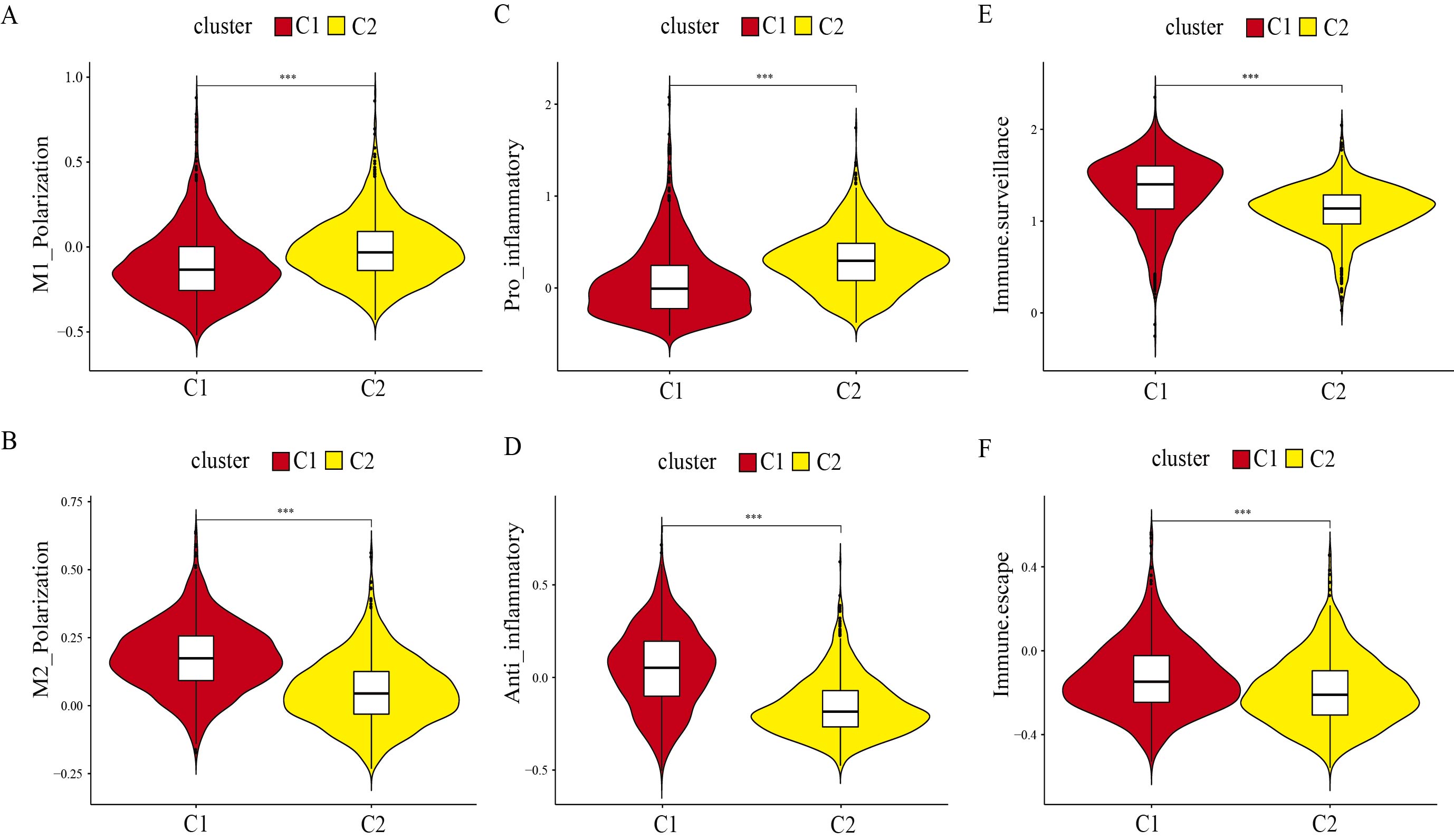

Supplement: Supplementary Figure 9 — Comparison of immune function between C1-Ma-C1QA and C2-Ma-THBS1 macrophages. Violin plots showing the AddModuleScore differences of M1-polarization (A), M2-polarization (B), pro-inflammatory (C), anti-inflammatory (D), immune surveillance (E), and immune escape (F) between C1-Ma-C1QA and C2-Ma-THBS1 macrophages. [file Image_9.jpeg]

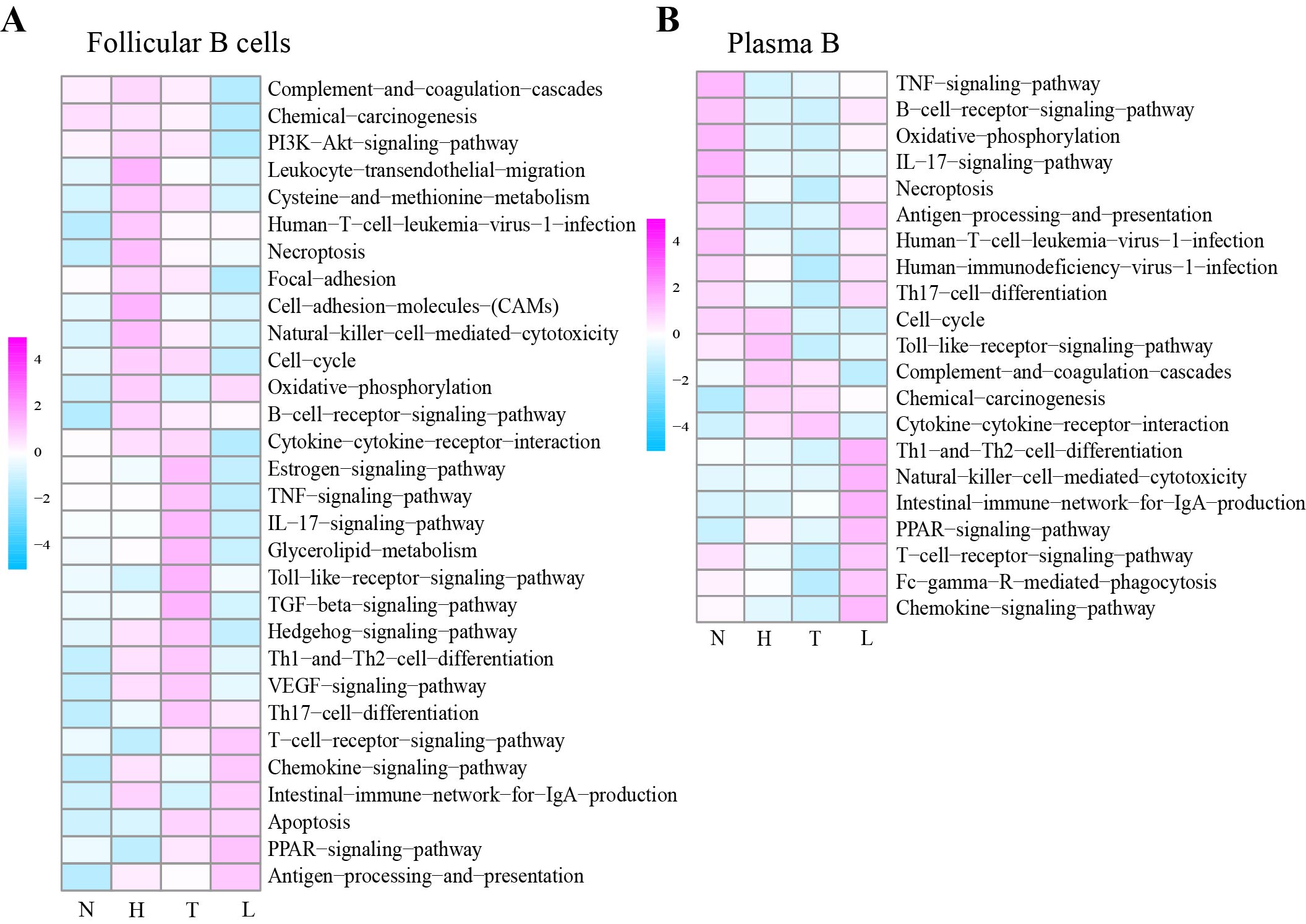

Supplement: Supplementary Figure 10 — The function of follicular B cells and plasma cells among four groups (N, H, T and L). Heatmap showing the enrichment of biological function in follicular B cells among four groups (A). Heatmap showing the enrichment of biological function in plasma cell among four groups (B). [file Image_10.jpeg]
